# Maize *Zmcyp710a8* Mutant as a Tool to Decipher the Function of Stigmasterol in Plant Metabolism

**DOI:** 10.3389/fpls.2021.732216

**Published:** 2021-11-03

**Authors:** Siddique I. Aboobucker, Lucas J. Showman, Thomas Lübberstedt, Walter P. Suza

**Affiliations:** ^1^Department of Agronomy, Iowa State University, Ames, IA, United States; ^2^W. M. Keck Metabolomics Research Laboratory, Iowa State University, Ames, IA, United States

**Keywords:** *Arabidopsis*, cell signaling, maize, metabolomics/metabolite profiling, stigmasterol, transcriptional regulation

## Abstract

Sterols are integral components of membrane lipid bilayers in eukaryotic organisms and serve as precursors to steroid hormones in vertebrates and brassinosteroids (BR) in plants. In vertebrates, cholesterol is the terminal sterol serving both indirect and direct roles in cell signaling. Plants synthesize a mixture of sterols including cholesterol, sitosterol, campesterol, and stigmasterol but the signaling role for the free forms of individual plant sterols is unclear. Since stigmasterol is the terminal sterol in the sitosterol branch and produced from a single enzymatic step, modifying stigmasterol concentration may shed light on its role in plant metabolism. Although *Arabidopsis* has been the model of choice to study sterol function, the functional redundancy of *AtCYP710A* genes and the presence of brassicasterol may hinder our ability to test the biological function of stigmasterol. We report here the identification and characterization of *ZmCYP710A8*, the sole maize C-22 sterol desaturase involved in stigmasterol biosynthesis and the identification of a stigmasterol-free *Zmcyp710a8* mutant. *ZmCYP710A8* mRNA expression pattern correlated with transcripts for several sterol biosynthesis genes and loss of stigmasterol impacted sterol composition. Exogenous stigmasterol also had a stimulatory effect on mRNA for *ZmHMGR* and *ZmSMT2*. This demonstrates the potential of *Zmcyp710a8* in understanding the role of stigmasterol in modulating sterol biosynthesis and global cellular metabolism. Several amino acids accumulate in the *Zmcyp710a8* mutant, offering opportunity for genetic enhancement of nutritional quality of maize. Other cellular metabolites in roots and shoots of maize and *Arabidopsis* were also impacted by genetic modification of stigmasterol content. Yet lack of obvious developmental defects in *Zmcyp710a8* suggest that stigmasterol might not be essential for plant growth under normal conditions. Nonetheless, the *Zmcyp710a8* mutant reported here is of great utility to advance our understanding of the additional roles of stigmasterol in plant metabolism. A number of biological and agronomic questions can be interrogated using this tool such as gene expression studies, spatio-temporal localization of sterols, cellular metabolism, pathway regulation, physiological studies, and crop improvement.

## Introduction

Sterols are important components of plasma membranes in eukaryotic organisms. They are also precursors for synthesis of steroid hormones such as testosterone, estrogen, glucocorticoids and mineral corticoids in mammals, ecdysteroids in insects and crustaceans, antheridiol and oogoniol (mating hormones of fungi), and BR in plants (Fujioka et al., 1997; Noguchi et al., 1999; Nomura et al., 1999; Friedrichsen and Chory, 2001; Bishop and Koncz, 2002; Clouse, 2002). Cholesterol is the major sterol in vertebrates, while fungi and some unicellular algae synthesize ergosterol as their principal sterol (Hartmann, 1998; Benveniste, 2002). In contrast, plants produce a mixture of sterols including cholesterol, sitosterol, stigmasterol, and campesterol, with sitosterol being the most abundant (Hartmann, 1998, 2004; Chappell, 2002; Clouse, 2002; Schrick et al., 2002; Lindsey et al., 2003; Schaller, 2003).

**FIGURE 1:**
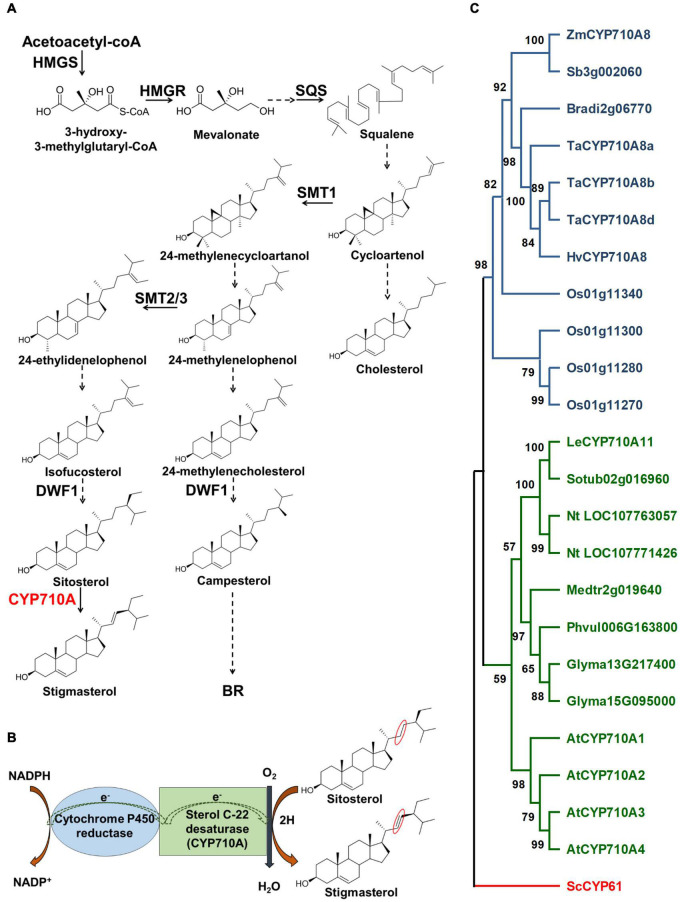
Stigmasterol biosynthesis and the phylogenetic relationship of CYP710A-like homologs. **(A)** The biosynthetic pathway of plant sterols leading to stigmasterol. Stigmasterol synthesis starts from acetate followed by a series of steps. Plants produce a mixture of sterols, in addition to stigmasterol, including campesterol (24-methyl) and sitosterol. The enzyme, sterol C-22 desaturase, catalyzes the conversion of sitosterol to stigmasterol. Campesterol is the preferred precursor of brassinosteroids (BR). Dashed arrows indicate multiple steps and solid arrows denote single step in the pathway. HMGS, 3-hydroxy-3-methylglutaryl-CoA synthase; HMGR, 3-hydroxy-3-methylglutaryl-CoA reductase; SQS, squalene synthase; SMT1, sterol methyltransferase 1; SMT2/3, sterol methyltransferase2/3; DWF1, C-24 sterol reductase; CYP710A, sterol C-22 desaturase. **(B)** A cartoon representation of the mechanism of sterol C-22 desaturase mediated conversion of sitosterol to stigmasterol. The site of desaturation is marked in red circles. **(C)** Phylogenetic relationship of the CYP710A-like homologs. Bootstrap values are shown as percentages (based on 1,000 replicates). *Saccharomyces cerevisiae* CYP61 was used to root the tree. Zm, *Zea mays*; Sb, *Sorghum bicolor*; Bradi, *Brachypodium distachyon*; Ta, *Triticum aestivum*; Hv, *Hordeum vulgare*; Os, *Oryza sativa*; Le, *Lycopersicum esculentum*; Sotub, *Solanum tuberosum*; Nt, *Nicotiana tabacum*; Medtr, *Medicago truncatula*; Phvul, *Phaseolus vulgaris*; Glyma, *Glycine max*; At, *Arabidopsis thaliana*; Sc, *Saccharomyces cerevisiae*.

The biosynthesis of plant sterols occurs primarily through the mevalonate (MVA) pathway (Figure 1A) and involves several enzyme-catalyzed steps (Goldstein and Brown, 1990; Chappell, 1995). Key enzymes in the MVA pathway including HMGS, HMGR, and SQS catalyze cytosolic reactions leading to the formation of squalene. Reactions beyond squalene synthesis occur in the ER producing cycloartenol in plants or lanosterol in vertebrates and yeast (Benveniste, 2002; Bach, 2016). Cycloartenol is a target of SMT1 to direct carbon flow toward the plant sterol (phytosterol) pathway (Diener et al., 2000; Sonawane et al., 2016). Consequently, the activities of SMT1 and SMT2/3 result in methylated sterols (campesterol and crinosterol) and ethylated sterols (sitosterol and stigmasterol), respectively (Schaeffer et al., 2001). Cycloartenol is also used by sterol side chain reductase (SSR) to synthesize cholesterol (Sawai et al., 2014; Sonawane et al., 2016). Downstream of SMT1 and SMT2/3, DWF1 contributes to both the stigmasterol and campesterol branches (Choe et al., 1999; Best et al., 2016), and campesterol is used to produce crinosterol and BR. Sitosterol can be transformed to stigmasterol *via* a reaction catalyzed by cytochrome P450 CYP710A sterol C-22 desaturase (Benveniste, 2002; Morikawa et al., 2006; Figure 1).

Work in *Arabidopsis* sterol mutants such as *fackel* and *hydra* revealed that BR application fails to rescue vascular patterning and embryonic defects, suggesting a direct signaling role for plant sterols (Jang et al., 2000; Schrick et al., 2000; Clouse, 2002; Souter et al., 2002; Lindsey et al., 2003; Nakamoto et al., 2015). This was further supported by studies involving *Arabidopsis* transcription factors belonging to a family of homeodomain-steroidogenic acute regulatory (StAR)-related lipid transfer (HD-START) proteins (Schrick et al., 2004, 2014). The START domain transcriptional factors include PHABULOSA and PHAVOLUTA, GLABRA2 and REVOLUTA/INTERFASICULAR FIBERLESS1 with roles in regulation of radial patterning, trichome and root hair development, and meristem initiation, respectively (Masucci et al., 1996; McConnell et al., 2001; Otsuga et al., 2001). Because the START domain in HD-START transcription factors contain a putative sterol/lipid binding motif, it has been postulated that a lipid or plant sterol may bind to modulate their function (McConnell et al., 2001; Schrick et al., 2004; reviewed by Fukuda, 2004). This raises another important question whether individual plant sterols might have specific signaling functions during plant growth and development. However, the lethal nature of mutations eliminating the production of campesterol and sitosterol makes it challenging to study their direct roles in signaling (Souter et al., 2002; Lindsey et al., 2003; Carland et al., 2010).

Stigmasterol is the terminal sterol in the sitosterol branch and produced from a single enzymatic step, and even though studies in *Arabidopsis* have suggested that stigmasterol loss might not be lethal (Morikawa et al., 2006), the *hydra/fackel* mutants that overproduce stigmasterol, display phenotypes resembling compromised START domain-containing genes (Lindsey et al., 2003). In addition, stigmasterol binds plasma membrane H^+^-ATPase and InteractoR Of SYnaptotagmin1 (ROSY1) involved in ion homeostasis and gravitropism, respectively (Grandmougin-Ferjani et al., 1997; Hartmann, 1998; Dalal et al., 2016; Aboobucker and Suza, 2019), and affects the expression of genes involved in cell expansion and growth (He et al., 2003). These observations suggest that stigmasterol might play an important function in plant growth and development.

Although *Arabidopsis* has been the model of choice to study sterol function, the challenge is the occurrence of multiple *AtCYP710A* genes. *AtCYP710A1* encodes the key enzyme for stigmasterol production, AtCYP710A2 and AtCYP710A4 also have low activity toward sitosterol, and AtCYP710A2 plays a role in producing brassicasterol (Morikawa et al., 2006; Arnqvist et al., 2008). The functional redundancy of *AtCYP710A* genes and the presence of brassicasterol may hinder our ability to decipher the direct function of stigmasterol in plant growth and development. In contrast, maize is rich in genomic and genetic resources and is predicted to encode a single sterol C-22 desaturase (Figure 1C; Aboobucker and Suza, 2019), making it an ideal system for studying the direct function of stigmasterol in plants. Moreover, identification of genes controlling stigmasterol production would benefit breeding maize varieties with favorable sterol composition for healthier seed oil (Ostlund, 2002).

In the present study, we utilized genetic, molecular, biochemical, and transgenic approaches to (i) identify the maize sterol C-22 desaturase, (ii) isolate stigmasterol-free maize and create *Arabidopsis* plants with altered stigmasterol concentration in roots and shoots, and (iii) demonstrate the potential of newly identified stigmasterol-free maize mutant in understanding the role of stigmasterol in sterol biosynthesis and global cellular metabolism.

## Materials and Methods

### Plant Materials and Growth Conditions

Maize inbred lines (B73, W22, Mo17, PH207) and expired Plant Variety Protection lines (PHB47, PHZ51) were from the North Central Regional Plant Introduction Station (USDA-ARS, Ames, IA, United States). Maize UniformMu transposon mutant stocks for *Zmcyp710a8-1* (mu1034077, UFMu-03461) and *Zmcyp710a8-2* (mu1019883, UFMu-00809) alleles were acquired from Maize Genetics Cooperation Stock Center (USDA-ARS, Urbana-Champaign, IL, United States). Transposon insertions were genotyped as previously described (Liu et al., 2016). For gene expression and sterol analysis of maize at V1 stage (Ritchie et al., 2005), seedlings were grown for 10 days in Sunshine LC1 mix (SunGro Horticulture, Agawam, MA, United States) in a growth chamber with light intensity 200 μmol m^–2^ s^–1^, 16:8 h day/night cycle and 25°C (day)/22°C (night). For mutant characterization and non-targeted metabolite analysis, maize seedlings were grown in cigar rolls as previously described (Kumar et al., 2012) for 11 days without fungicide. Tissue samples were frozen immediately in liquid nitrogen and stored in –80°C.

*Arabidopsis thaliana* var. Columbia (Col-0, stock CS70000) seeds were from Arabidopsis Biological Resource Center (Columbus, OH) and the *Atcyp710a1* mutant (GK-325E09, stock N421631; Wang et al., 2012) seeds were from Nottingham Arabidopsis Stock Center (Loughborough, United Kingdom). *Arabidopsis* was grown in the following conditions: light intensity was 150 μmol m^–2^ s^–1^, 16:8 h day/night cycle and 23°C. Seeds were grown in soil for plant transformation, transformants identification, and sterol analysis. For non-targeted metabolite analysis, surface-sterilized *Arabidopsis* seeds were grown in Magenta boxes as described by Suza and Staswick (2008) in liquid Murashige and Skoog (MS) medium (Murashige and Skoog, 1962). Tissues from 3-week-old seedlings were harvested, frozen in liquid nitrogen and stored in –80°C.

### Chemicals and Reagents

Stigmasterol, ribitol, nonadecanoic acid, methoxyamine hydrochloride, pyridine, and 5α-cholestane were obtained from Sigma-Aldrich (St. Louis, MO, United States), sitosterol from Avanti Polar Lipids (Alabaster, AL, United States), campesterol and brassicasterol were from Cayman chemicals (Ann Arbor, MI, United States). Glufosinate ammonium was from Crescent Chemicals (Islandia, NY, United States). Chloroform, methanol, diethyl ether, hexane, and HPLC water were obtained from Fisher Scientific (Fair Lawn, NJ, United States).

### Phylogenetic Analysis

Phylogenetic analysis using the Maximum Likelihood method and JTT matrix-based model (Jones et al., 1992) was carried out to evaluate the evolutionary relationship of Zm00001d039384 with other CYP710A-like homologs. Full length protein sequences were aligned using MUSCLE algorithm (Edgar, 2004). The tree construction was carried out with default parameters: 1,000 bootstrap replicates and, uniform sites and use all sites in MEGA X (Kumar et al., 2018). *Saccharomyces cerevisiae* CYP61 was used to root the tree. The protein sequences were from previous reports or downloaded from the Phytozome portal^1^ and are as described previously (Aboobucker and Suza, 2019).

### Vector Construction and *Arabidopsis* Transformation

*ZmCYP710A8* ORF was amplified from maize B73 genomic DNA using primer pairs, ZmCYP710A8_F and ZmCYP710A8_R (Supplementary Table 1). PrimeSTAR GXL DNA Polymerase (Clontech, Mountain View, CA, United States) was used with the following conditions: 98°C for 10 sec initial denaturation followed by 35 cycles of 98°C for 10 s, 55°C for 15 s and 68°C for 90 s and a final 68°C for 5 min. After purification using PCR purification kit (Qiagen, Germantown, MD, United States), the purified PCR product and pTF101.1-35S vector were digested with *Kpn*I and *Sac*I (New England Biolabs, Ipswich, MA). After ligation for overnight at 4°C, they were transformed into NEB10-b cells. Successful clones were transformed into *Agrobacterium tumefaciens* strain C58C1 using the freeze-thaw method (Holsters et al., 1978).

*Arabidopsis* transformation was using the floral dip method (Clough and Bent, 1998). Putative transformants were identified by spraying glufosinate (60 mg/L) at stage 1.02 (Boyes et al., 2001) and PCR confirmation. Single insertion and homozygous lines based on Mendelian segregation were identified by growing seedlings *in vitro* with 10 mg/L glufosinate.

### Hormone Treatment

Hormone treatment method was adapted from Ortiz-Masia et al. (2007) and Louis et al. (2015). Hormone stock solutions (1000×) of methyl jasmonate (50 mM), abscisic acid (100 mM), indole acetic acid (100 mM) or salicylic acid (50 mM) were prepared fresh every time by dissolving in ethanol. To prepare 10 mM 6-benzyladenine stock solution, it was first dissolved in 2.5 mL (2.5 M HCl) followed by adding 2.5 mL ethanol to make a total of 5 mL. Final hormone spraying solutions (400 mL) were prepared by diluting freshly made stock solutions (or ethanol for mock) in double-distilled water + 0.1% Tween-20. Ten days old (V1 stage; Ritchie et al., 2005) maize B73 seedlings (3 per pot) were sprayed with a fine mist of the hormone solutions until drip around mid-day (11 AM). Second leaf samples (Figure 3A; Yan et al., 2015) from three seedlings were pooled and frozen immediately in liquid nitrogen for one treatment per time point. All samples were stored in –80°C until further processing.

### Exogenous Sterol Treatment

Sterol stock solutions were prepared by dissolving sterol powders in ethanol and stored in –20°C. For gene expression experiments, feeding solutions of sterols (10 μM in ddH_2_O) were prepared by diluting stock solutions one day prior and equilibrated in the growth chamber. New germination papers were soaked by spraying with the feeding solution or ddH_2_O (control). 4- or 11-days old maize seedlings were placed on the papers and rolled in such a way that roots are completely covered inside the roll, while shoots protrude from the rolls. Feeding solutions were also poured over the rolls at the shoot-root junction to ensure the rolls were sufficiently drenched. The rolls were then placed in beakers containing feeding solutions, while ensuring that only the bottom ∼5 cm of the rolls (of the 30 cm long) was immersed in the solutions. For non-targeted metabolite analysis, the feeding procedure was the same except for two modifications. First, stigmasterol (5 μM) was used and second, maize seedlings were 4 days old when the feeding was performed and incubated for another 7 days. After the indicated times, root or shoot tissues were harvested, frozen in liquid nitrogen, and stored at –80°C.

### RNA Extraction and qRT-PCR Analysis

Total RNA from maize or *Arabidopsis* was extracted using RNeasy Plant Mini Kit (Qiagen, Germantown, MD, United States) and treated with rDNaseI (Thermo Fisher, Waltham, MA, United States) per manufacturers’ instructions. First strand cDNA was synthesized from 1 μg of total RNA and oligo (dT)_20_ primers using Superscript III First Strand cDNA synthesis system (Invitrogen, Carlsbad, CA, United States). For qRT-PCR analysis, SsoAdvanced Universal SYBR Green Supermix (Bio-Rad, Hercules, CA, United States) was used on an Applied Biosystems Mx3000P thermocycler with 10 ng (for maize) or 5 ng (for *Arabidopsis*) of cDNA as template in a 10 μL reaction. Primer sequences are described in Supplementary Table 1. Relative expression levels compared to internal reference *ZmACTIN* (Louis et al., 2015) or *AtEF1α-A* (Aboobucker et al., 2017) were calculated using the 2^–ΔΔCt^ method (Pfaffl, 2001).

### Sterol Analysis

Sterol analysis was performed as previously described (Suza and Chappell, 2016) with following modifications. Lyophilized tissue powder (∼20 mg) and 8 μg 5α-cholestane (internal standard) were added in a clean glass tub together with chloroform (5 mL) and sonicated at room temperature (RT) for 10 min followed by a 45 min incubation at 50°C. After equilibrating to RT, water (5 mL) was added, vortexed and incubated at 50°C for 45 min. The organic layer was transferred to a new tube and dried under dry nitrogen. The samples were re-suspended in chloroform (2 mL), hydrolyzed with 1 mL of 1.25 M HCl (in methanol) and incubated at 50°C for 2 h. The samples were dried under nitrogen followed by methanol (1 mL) addition and dried under nitrogen. Organic extracts were re-suspended in 1.2 mL of diethyl ether:hexane (9:1, v/v) and transferred to a new vial followed by drying under nitrogen. The samples were derivatized in 60 μL pyridine and 40 μL BFTSA + 1% TMCS (Sigma) and incubating at 50°C for 1 h (Koek et al., 2006).

Trimethylsilyl derivatized samples were analyzed using an Agilent Technologies Gas Chromatograph (Model 7890C) equipped with a DB-5 column (Agilent, 30 m × 0.25 mm, 0.25 mm phase thickness) and coupled to a mass spectrometer (Agilent, Model 5975C). The samples were injected in split less mode with an inlet and transfer line temperature of 280°C. Helium at a flow rate of 1 mL/min was the carrier gas. The initial oven temperature was 110°C and subsequently increased to 275°C at a gradient of 20°C/min followed by a gradient of 2°C/min up to 320°C held for 6 min. The mass spectrometer was held at standard settings with an auto tune method used for calibrating and tuning. Quantitative sterol measurements were by integrating peak areas to each sterol species and comparing to the internal standard peak area. Peak detection and deconvolution were performed using AMDIS (Automated Mass Spectral Deconvolution and Identification System, National Institute of Standards and Technology) software. Peaks were identified by comparing to available spectra in NIST17/Wiley11 libraries and authentic standards and a previous report for steryl glucosides (Phillips et al., 2005; Supplementary Figure 1).

### Mass Spectrometry Imaging of Sterols

Roots of 3-day-old maize seedlings were embedded in cryo-molds containing 2% medium viscosity carboxymethylcellulose (CMC; Sigma-Aldrich, St. Louis, MO, United States). The embedded roots were immediately placed in a prechilled cryostat (Leica Biosystems, Buffalo Grove, IL) to equilibrate prior to being cryosectioned. 30 μm thick longitudinal sections of CMC embedded roots were taken and applied directly onto conductive indium tin oxide (ITO) coated slides (part no.: 8237001, Bruker Daltonik, Bremen, Germany). Sample sections were dried and brought to room temperature in a vacuum desiccator. Prior to matrix deposition, bright field images of the root sections were captured using a Macro Zoom imaging system (ZEISS, Oberkochen, German). A matrix consisting of sputter coated silver was applied to the root sections for 30 s using a Denton Desk II sputter coater (Denton Vacuum, Moorestown, NJ, United States) equipped with a silver target (99.99% Ag, Ø60 mm × 0.1 mm) (Ted Pella, Inc., Redding, CA, United States).

Mass spectrometry imaging (MSI) was performed using a Bruker SolariX fourier-transform ion cyclotron resonance mass spectrometer (FT-ICR MS) equipped with a 7.0 tesla superconducting magnet (Bruker Daltonik, Bremen, Germany). Sterols, as silver cationic adducts, were detected using matrix-assisted laser desorption/ionization mass spectrometry (MALDI-MS), as described previously (Jun et al., 2010; Dufresne et al., 2013; Sekula et al., 2015; Xu et al., 2015). MSI data was acquired in positive mode with a mass range from m/z 200.90 to 2000 while collecting one Megaword of data points per scan. A quadrupole isolation ranging from m/z 500-530 was used to isolate the sterol adducts and reduce silver aggregate background interference. The laser raster was set to acquire 50 μm spots. The FT-ICR MS instrument was operated using ftmsControl software (version 2.1 version 4.1, Bruker Daltonik, Bremen, Germany) while flexImaging software (version 4.1, Bruker Daltonik, Bremen, Germany) was used to collect and analyze the imaging data.

### Non-targeted GC/MS Metabolite Analysis

#### Extraction

Non-targeted metabolites were extracted from maize and *Arabidopsis* samples according to previously published methods with modifications (Fiehn et al., 2000). Briefly, ∼10 mg of lyophilized tissue powder of maize or *Arabidopsis* samples was used. Internal standards were added as follows: Ribitol (1 mg/mL; polar standard) 10 μL for *Arabidopsis* root, 20 μL for shoot and 35 μL for maize root and shoot. Nonadecanoic acid (1 mg/mL; non-polar standard) was added at 10 μL for *Arabidopsis* root, 20 μL for shoot and 12 μL for maize root and shoot. Hot methanol (60°C) was added and vortexed for 2 min and incubated at 60°C for 10 min. Samples were sonicated at RT for 10 min using a sonicating water bath (Danbury, CT) followed by chloroform (0.35 mL) and vortexing for 2 min. Next, sterile water (0.3 mL) was added and vortexed for 2 min followed by centrifugation for 9 min at maximum speed in a tabletop centrifuge. The polar and non-polar phases were transferred to separate glass vials and dried in a speed-vac for overnight.

#### Derivatization

A two-step derivatization procedure involving methoximation and silylation was done. First, 50 μL of methoximation mixture (freshly prepared by dissolving 20 mg methoxyamine hydrochloride in 1 mL pyridine) were added to the vial, vortexed and reacted for 90 min at 30°C. Silylation was performed next by adding 70 μL of BSTFA + 1% TMCS and incubating at 60 °C for 30 min (Koek et al., 2006) and cooled to RT before GC-MS analysis.

#### GC-MS Analysis

Agilent Technologies Gas Chromatograph (Model 7890C) equipped with a DB-1 column (Agilent: 122-0112, 15 m × 250 μm × 0.25 μm phase thickness) and coupled to a mass spectrometer (Agilent; Model 5975C) was used. Polar and non-polar fractions of samples were injected individually in split less mode with an inlet temperature of 310°C and transfer line temperature of 280°C. Helium at a flow rate of 1.2 mL/min was the carrier gas. The initial oven temperature was 70°C held for 0.5 min and increased at a gradient of 12°C/min to 340°C held for 10 min. The mass spectrometer was operated at a source temperature of 280°C and standard settings with an autotune method.

#### Data Analysis

Peak detection and deconvolution were performed using AMDIS software. Peaks were identified by comparing to spectra and retention index data in NIST17/Wiley11 libraries. Abundances were calculated relative to the internal standards and individual sample mass. Features with at least 90% values were maintained and for those with missing values were imputed using k-nearest neighbors (KNN) in MetaboAnalyst (Xia et al., 2009). Fold changes were calculated to the relevant wild-type control samples. The ANOVA analysis tool was used to find and visualize significant features in MetaboAnalyst (Xia et al., 2009) with a *p*-value < 0.05 and an FDR < 0.3. For maize, features correlating with stigmasterol were filtered from ANOVA results. Heatmaps were generated using Microsoft Excel 365.

### Statistical Analysis

Statistical analyses were conducted as described in the figure or table legends. *P* values were calculated by two-tailed Student’s *t-*test using Microsoft 365 Excel or by one-way ANOVA multiple comparisons with Tukey test in R.

## Results

### Identification of a Maize Sterol C-22 Desaturase Involved in Stigmasterol Biosynthesis

Identification of putative sterol C-22 desaturases in maize involved querying the MaizeGDB database^2^ with *At*CYP710A1 amino acid sequence (Morikawa et al., 2006). The query resulted in a single 1,551 bp intron-less gene model (*Zm00001d039384*) predicted to encode a 515 aa protein with 63% similarity to *At*CYP710A1 and we renamed it to *ZmCYP710A8* as per P450 nomenclature (Nelson, 2009). Multiple sequence alignment showed that the amino acid sequence of *Zm*CYP710A8 is highly similar to CYP61 from yeast, and previously characterized CYP710A proteins from plants (Supplementary Figure 2). Further, conserved sites and characteristic motifs for CYP710A are also present in *Zm*CYP710A8 (Supplementary Figure 2).

Phylogenetic analysis suggested that other grass species may also encode single sterol C-22 desaturases (Figure 1C). The CYP710A-like homologs cluster into two monophyletic clades, monocots, and eudicots, with *Zm*CYP710A8 placed in the monocot clade. Moreover, no CYP710A-like gene from either clade clustered together, suggesting that the plant CYP710A-like genes are from a single ancestor and developed into different branches after the lineages diverged. Within the clades, some species have more than one representative, suggesting these paralogs might have resulted from recent genome duplication events. These findings suggested that *ZmCYP710A8* may encode the maize sterol C-22 desaturase.

**FIGURE 2:**
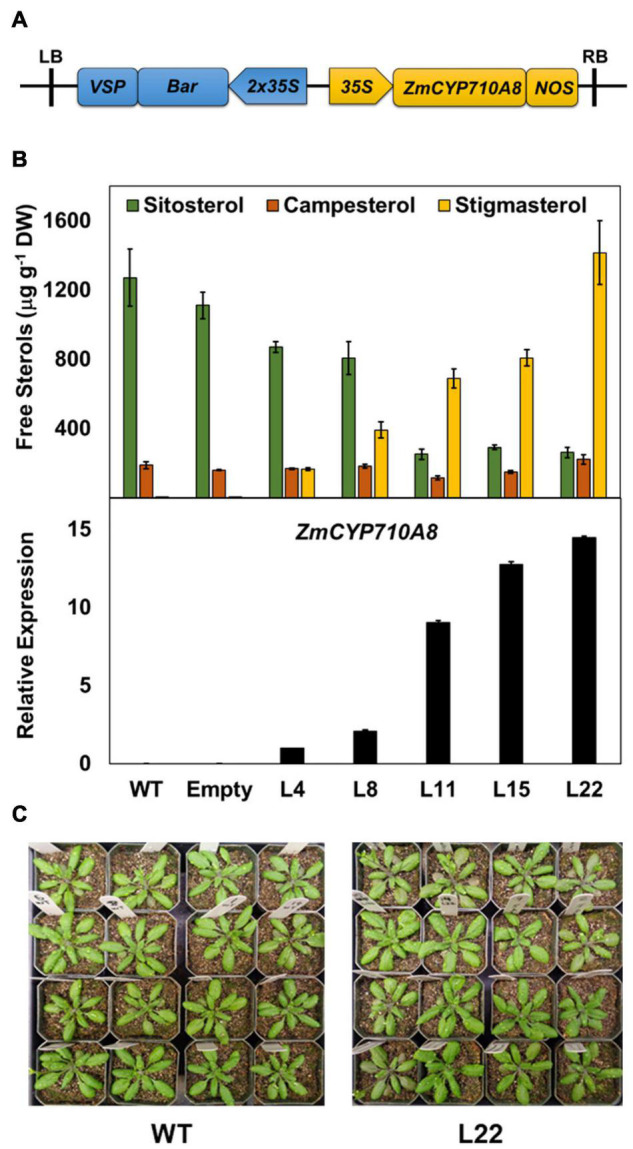
Heterologous expression of *ZmCYP710A8* in *Arabidopsis* increases stigmasterol at the expense of sitosterol. **(A)** Schematic of the T-DNA region of the molecular construct used to overexpress the presumed coding region of *ZmCYP710A8* sequence in *Arabidopsis thaliana* Col-0 background. LB, left border; RB, right border; 35S, cauliflower mosaic virus constitutive promoter; Bar, phosphinothricin N-acetyltransferase gene as selectable marker; VSP, vegetative storage protein terminator; NOS, nopaline synthase terminator. **(B)** Free major sterol contents of transgenic *Arabidopsis thaliana* rosette leaves expressing the maize gene *ZmCYP710A8* in Col-0 background. Also shown, in the bottom panel, is the quantification of *ZmCYP710A8* mRNA in the rosette leaves relative to *AtEF1α-A* (used as a reference gene for normalization). WT, Col-0 wild type; Empty, Col-0 transformed with empty vector; L4, L8, L11, L15, and L22 are five independent transgenic events. Tissue samples were pooled from rosette leaves of 10 individual plants (4-week-old) for each line, split to do both sterol and mRNA analysis. Sterol measurements are shown as means ± SD from four technical replicates. mRNA measurements are shown as means ± SD from three technical replicates. **(C)** Images of 4-week-old wild type and a *ZmCYP710A8* overexpressor (L22) *Arabidopsis* plants are shown.

To validate the function of *ZmCYP710A8* in stigmasterol biosynthesis, the full-length coding sequence was transformed into *Arabidopsis* (Figure 2A) and positive transformants were confirmed by PCR. Sterols were analyzed by GC-MS from rosette leaves of transgenic *Arabidopsis* overexpressing *ZmCYP710A8* with single insertion in the T3 generation (Supplementary Data 1). Wild-type (Col-0) produced trace amounts of stigmasterol, while its concentration in transgenic lines ranged from 166.3 to 1415.2 μg g^–1^ DW (Figure 2B). The stigmasterol increase in transgenic plants was concomitant with a reduction in sitosterol, suggesting a direct substrate-product relationship. Further, stigmasterol content strongly correlated with *ZmCYP710A8* mRNA levels (Figure 2B), suggesting transcriptional regulation of stigmasterol biosynthesis. The concentration of campesterol, however, remained relatively constant in transgenic lines. The apparent morphology of *ZmCYP710A8* overexpressing *Arabidopsis* plants did not differ from that of wild type plants (Figure 2C). Taken together, *ZmCYP710A8* encodes a sterol C-22 desaturase in maize.

**FIGURE 3:**
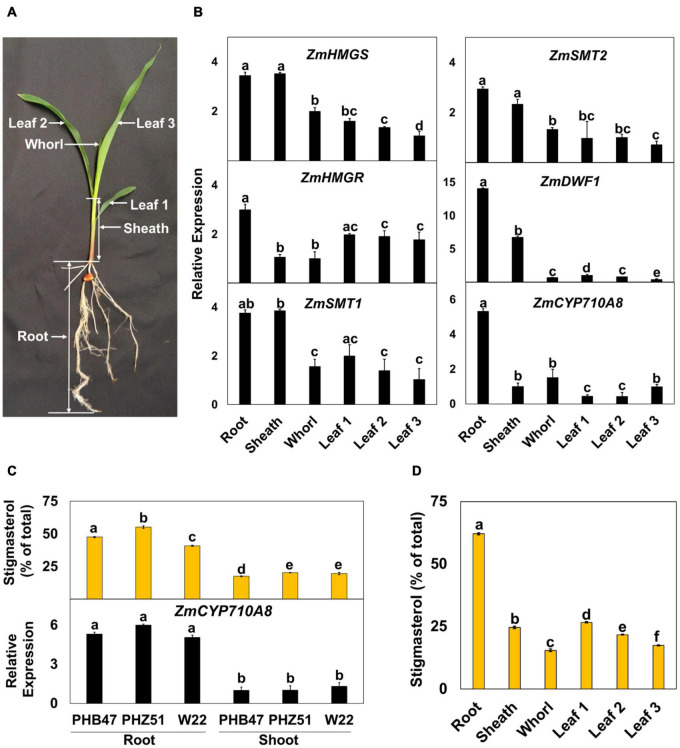
Expression of mRNAs for *ZmCYP710A8* and sterol biosynthesis genes correlate with stigmasterol composition during maize development. **(A)** A representative B73 seedling at the V1 stage and the different organs used in the analysis are shown. **(B)** Relative mRNA levels of different sterol biosynthesis genes. Tissue samples were pooled from 3 individual seedlings in one experiment. mRNA quantification was done in three replicates. *ZmACTIN* was used as a reference gene for normalization. Data are means ± SE from two independent experiments. **(C)** Stigmasterol composition (% of total sterols) and quantification of *ZmCYP710A8* mRNA in different maize genotypes. Tissue samples for mRNA and sterol analysis are as described in panel **(B)**. Root is as shown in panel **(A)** and shoot is a pool of all the above ground tissue shown in panel **(A)**. Data are means ± SE from two independent experiments. **(D)** Stigmasterol composition (% of total sterols) of the different organs of maize B73 at V1 stage is shown. Tissue samples are as described in (b). Sterols were measured in four technical replicates for each experiment. Data are means ± SE from two independent experiments. One-way ANOVA was performed at a 95% confidence interval. Means that do not share a letter were significantly different.

### Expression Pattern of *ZmCYP710A8* During Development and Hormone Treatments

To investigate the developmental expression of *ZmCYP710A8*, we measured its mRNA in various tissues of maize seedlings (Figure 3A), along with mRNAs for several key plant sterol biosynthesis genes including *HMGS, HMGR, SQS, SMT1, SMT2* (Suza and Chappell, 2016). Except for *DWF1* (Best et al., 2016) and *ZmCYP710A8*, maize encodes multiple copies for *HMGS, HMGR, SQS, SMT1*, and *SMT2* (Supplementary Table 2). Therefore, PCR primers were designed to capture all molecular transcripts from a given gene family as previously described in *N. benthamiana* (Suza and Chappell, 2016).

Sterol profile of maize seedlings was also measured in addition to mRNA expression (Figure 3 and Table 1). Stigmasterol content was highest in roots compared to aerial tissues in B73 (Figure 3D) and a similar trend was observed in roots and shoots of additional maize genotypes (Figure 3C and Supplementary Figure 3). In contrast, sitosterol concentration displayed an inverse pattern than that of stigmasterol. Like stigmasterol, total sterol content was highest in roots followed by aerial tissues (Table 1). The mRNA levels for *ZmCYP710A8* and other sterol biosynthesis genes were highest in roots compared to aerial tissues (Figure 3B), correlating with stigmasterol content (Figure 3D). In addition, *ZmCYP710A8* mRNA levels strongly correlated (*R*^2^ = 0.94) with stigmasterol but showed an inverse correlation (*R*^2^ = –0.92) with sitosterol in B73 and other maize genotypes (Figure 3C, Supplementary Figure 3, Supplementary Table 3). The results suggest that the control of stigmasterol biosynthesis is at the transcriptional level (Suza and Chappell, 2016), and as suggested (Figure 2B), a single sterol C-22 desaturase may exist in the maize genome.

**TABLE 1:** Free sterols content (μg g^–1^ DW) in the various maize (B73) organs at V1 stage.

Tissue	Cholesterol	Campesterol	Sitosterol	Stigmasterol	Total
**Root**	Trace	584.02 ± 19.44	492.38 ± 32.97	1759.72 ± 58.47	2837.88 ± 109.92
**Sheath**	Trace	452.29 ± 13.12	1215.47 ± 42.99	544.26 ± 12.59	2213.28 ± 65.02
**Whorl**	Trace	242.55 ± 8.84	1430.92 ± 98.71	304.83 ± 10.00	1981.18 ± 115.29
**Leaf 1**	Trace	299.50 ± 7.61	720.59 ± 29.16	345.48 ± 11.03	1296.13 ± 47.37
**Leaf 2**	Trace	222.56 ± 8.68	782.48 ± 32.96	278.48 ± 9.95	1285.09 ± 51.70
**Leaf 3**	Trace	240.16 ± 7.09	1081.26 ± 40.1	278.72 ± 7.06	1601.29 ± 54.23

*Tissue samples were pooled from 3 individual seedlings in one experiment.*

*Sterols were measured in 4 technical replicates for each experiment.*

*Data are means ± SE from two independent experiments.*

**FIGURE 4:**
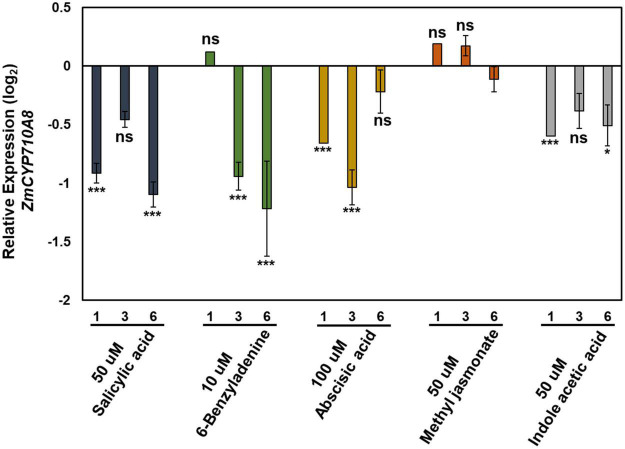
Relative expression of *ZmCYP710A8* mRNA in response to hormone treatments. Relative mRNA levels (log_2_) of *ZmCYP710A8* compared to mock in response to various hormone treatments for indicated hours after treatment. Leaf tissues (leaf 2) from three plants were pooled for one time point (as indicated) per treatment. mRNA levels were measured in triplicates with *ZmACTIN* was used as a reference gene for normalization. Data are means ± SE from two independent experiments. Asterisks indicate statistical significance by Students *t*-test compared to mock samples at their respective time points. *P < 0.05; ***P < 0.001; ns, not significant.

The expression pattern of *ZmCYP710A8* in response to phytohormone treatments was examined to study stigmasterol regulation by hormone treatment. All hormone treatments used except methyl jasmonate suppressed the expression of *ZmCYP710A8* at indicated time points and concentrations (Figure 4).

### *ZmCYP710A8* Is the Sole Maize Sterol C-22 Desaturase Involved in Stigmasterol Biosynthesis

**FIGURE 5:**
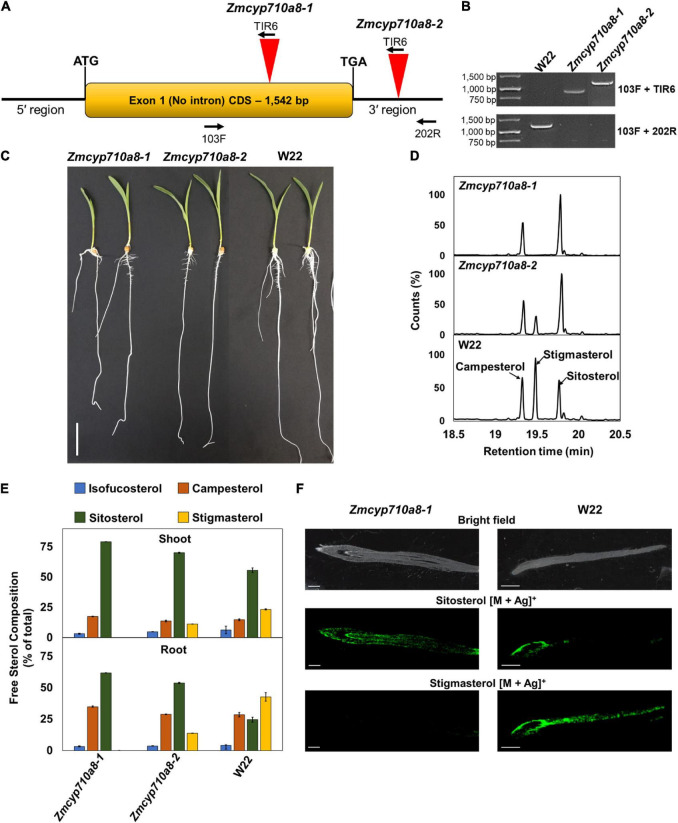
*ZmCYP710A8* is the sole maize C-22 sterol desaturase to synthesize stigmasterol. **(A)** Schematic of the *ZmCYP710A8* gene structure with its 5′ and 3′ UTRs. The coding sequence is shown as a yellow box with the start (ATG) and stop (TGA) codons marked. Inverted red triangles show the site of transposable element insertions in *Zmcyp710a8-1* and *Zmcyp710a8-2* mutant alleles. The primer binding sites used for genotyping the mutations are indicated with 103F, 200R, and TIR6. **(B)** Identification of transposon insertion in the *Zmcyp710a8* mutant alleles by PCR genotyping. The top panel shows PCR reactions amplified using one primer binding to the transposon and the other to the genome and the bottom panel shows amplicons from both primers binding to the genome. Transposon insertion would not lead to amplification of wild type band in mutants. Primer pairs are as shown in panel **(A)**. W22 is the wild type control. DNA ladder is also shown. **(C)** Representative images of 11-day old wild type and mutant maize seedlings grown by cigar roll method. Scale bar = 5 cm. **(D)** Representative GC-MS total ion chromatogram traces of root tissues from *Zmcyp710a8-1*, *Zmcyp710a8-2* mutant alleles and wild type (W22) are shown. The three major sterols are indicated. **(E)** The composition (% of total sterols) of free major sterols in shoot and root of the two mutant alleles and wild type are shown. Tissue samples were pooled from 3 individual seedlings in one experiment. Sterol extraction was performed for two independent experiments and measured in four technical replicates for each experiment and the data shown are means ± SD. Root is as shown in panel **(C)** and shoot is a pool of all the above ground tissue shown in panel **(C)**. **(F)** Mass spectrometry images of sterols in roots of 3-day-old maize seedlings. Root tip end is on the left side of images. The monoisotopic mass for each sterol were monitored with both major isotopes of silver (^107^Ag and ^109^Ag). The MS images are of sitosterol silver adducts (*m/z* 523.2913 and 521.2913) and stigmasterol silver adducts (*m/z* 519.2856 and 517.2865). Respective bright field images are included as the special reference for the MSI data. MSI images were captured using a 50 μm raster size. The images are representative of two experiments. Scale bar = 1 mm.

To generate a stigmasterol free system for studying its biological function in maize, we sought to identify plants compromised in stigmasterol production from the maize UniformMu population (McCarty et al., 2005). PCR analysis led to the identification of transposon insertions in two independent alleles (*Zmcyp710a8-1* and *Zmcyp710a8-2*) within and near the *ZmCYP710A8* open-reading frame (Figures 5A,B). *Zmcyp710a8* plants did not show any observable phenotypic defects compared to wild type and they were able to grow and produce seeds under controlled conditions and in the field (Figure 5C and Supplementary Figure 4).

Sterol analysis by GC-MS showed the *Zmcyp710a8-1* allele did not produce stigmasterol, but *Zmcyp710a8-2* produced 0.3- to 0.5-fold stigmasterol as the W22 wild type (Figure 5D and Supplementary Table 4). Compared to W22, the F_1_ progeny from a cross between W22 and *Zmcyp710a8-1* (W22/*Zmcyp710a8-1*) produced 0.8- and 0.6-fold less stigmasterol in roots and shoots, respectively (Supplementary Figure 5). These results support our phylogenetic analysis and heterologous expression data (Figures 1, 2) allowing us to conclude that *ZmCYP710A8* is the sole sterol C-22 desaturase in maize.

The composition of free sterols and their intermediates were altered in the *Zmcyp710a8-1* and *Zmcyp710a8-2* mutants. For instance, intermediate sterols including cycloartenol and isofucosterol were reduced in roots and shoots of *Zmcyp710a8-2* relative to W22 (Supplementary Table 4). In roots, sitosterol was 25% of the total sterols in W22, 54% in *Zmcyp710a8-2*, and 62% in *Zmcyp710a8-1*, while in shoots, it was 56% in W22, 70% in *Zmcyp710a8-2*, and 80% in *Zmcyp710a8-1* (Figure 3C and Supplementary Table 4).

Further, campesterol composition was significantly different between W22 and *Zmcyp710a8-1;* the difference in roots being 28% of total sterols in W22 and 35% in shoots, while in shoots W22 had 14% and *Zmcyp710a8-1* had 18%. There was no appreciable difference, however, in campesterol proportions between W22 and *Zmcyp710a8-2* (Figure 5E and Supplementary Table 4). Nonetheless, it is noteworthy that the composition of steryl glucosides was proportional to those of free sterols in roots and shoots (Supplementary Figure 6). Mass spectrometry imaging of wild type and *Zmcyp710a8-1* roots indicate that sitosterol is localized to the root tip region while stigmasterol is more broadly distributed (Figure 5F). Taken together, the disruption of *ZmCYP710A8* eliminates stigmasterol production and affects the composition of free sterols and steryl glucosides, and the distribution of sitosterol in roots.

### Exogenous Stigmasterol Affects Sterol Gene Transcripts

The positive correlation between stigmasterol content and mRNA for key sterol biosynthesis genes (Figure 3) suggests stigmasterol may have a stimulatory effect on gene expression. Identification of a stigmasterol-free background in *Zmcyp710a8-1* (Figure 5) provided a system to test whether genes such as *HMGR* and *SMT* whose expression in various tissues correlates with stigmasterol content (Figure 3) might be responsive to exogenously supplied stigmasterol.

**FIGURE 6:**
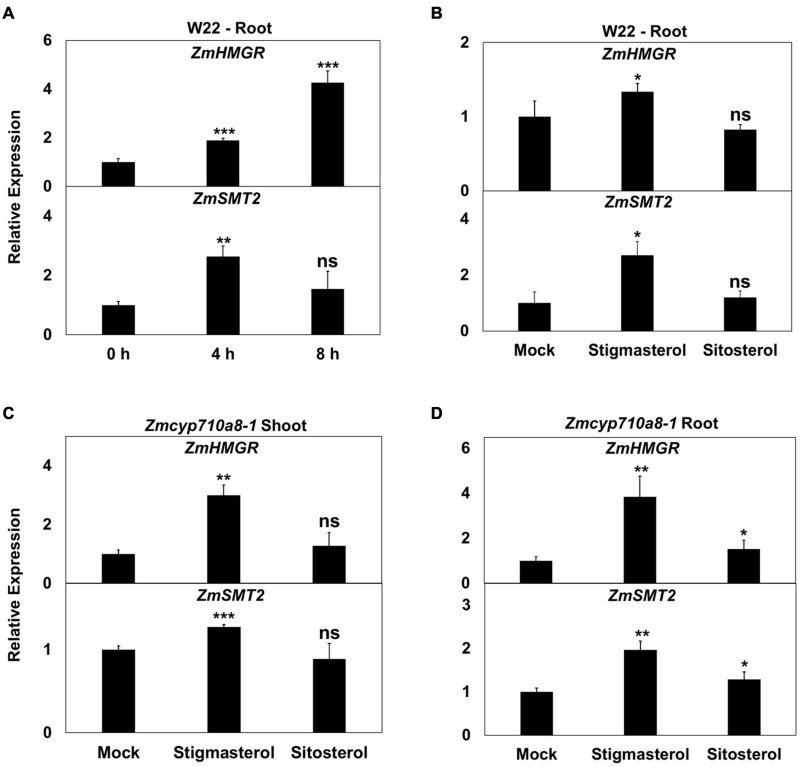
Impact of exogenous stigmasterol on the expression of maize sterol biosynthesis genes. **(A,B)** Relative mRNA quantification of *ZmHMGR* and *ZmSMT2* genes in wild type (W22) **(A)** in response to feeding 10 μM stigmasterol at different time points, **(B)** in response to 10 μM stigmasterol and 10 μM sitosterol feeding for 4 h. **(C,D)** Relative mRNA quantification of *ZmHMGR* and *ZmSMT2* genes in *Zmcyp710a8-1* background in response to various sterols (10 μM) for 4 h **(C)** in roots and **(D)** in shoots. *ZmACTIN* was used as a reference gene for normalization. Data are means ± SD. Asterisks indicate statistical significance by Students *t*-test compared to 0 h in panel **(A)** and to Mock in panels **(B,C,D)**. *P < 0.05; **P < 0.01; ***P < 0.001; ns, not significant.

Treatment of W22 roots with exogenous stigmasterol resulted in the induction of *ZmHMGR* and *ZmSMT2* mRNA after 4 and 8 h (Figure 6A). Although *ZmHMGR* mRNA remained elevated, *ZmSMT2* mRNA slightly decreased after 8 h (Figure 6A). Exogenous sitosterol, in contrast, did not have a significant impact on *ZmHMGR* and *ZmSMT2* mRNA after 4 h (Figure 6B) and 8 h (not shown).

Similar to the observation in W22, *ZmHMGR* and *ZmSMT2* mRNA levels were higher in roots and shoots of *Zmcyp710a8-1* treated with stigmasterol (Figures 6C,D). Sitosterol, however, had a modest effect on *ZmHMGR* and *ZmSMT2* mRNA in roots of *Zmcyp710a8-1* (Figures 6C,D). The impact of stigmasterol on mRNA for *ZmHMGR* and *ZmSMT2* suggests that stigmasterol might modulate transcripts for plant sterol biosynthesis genes.

### Stigmasterol Impacts Global Cellular Metabolites in Maize

**FIGURE 7:**
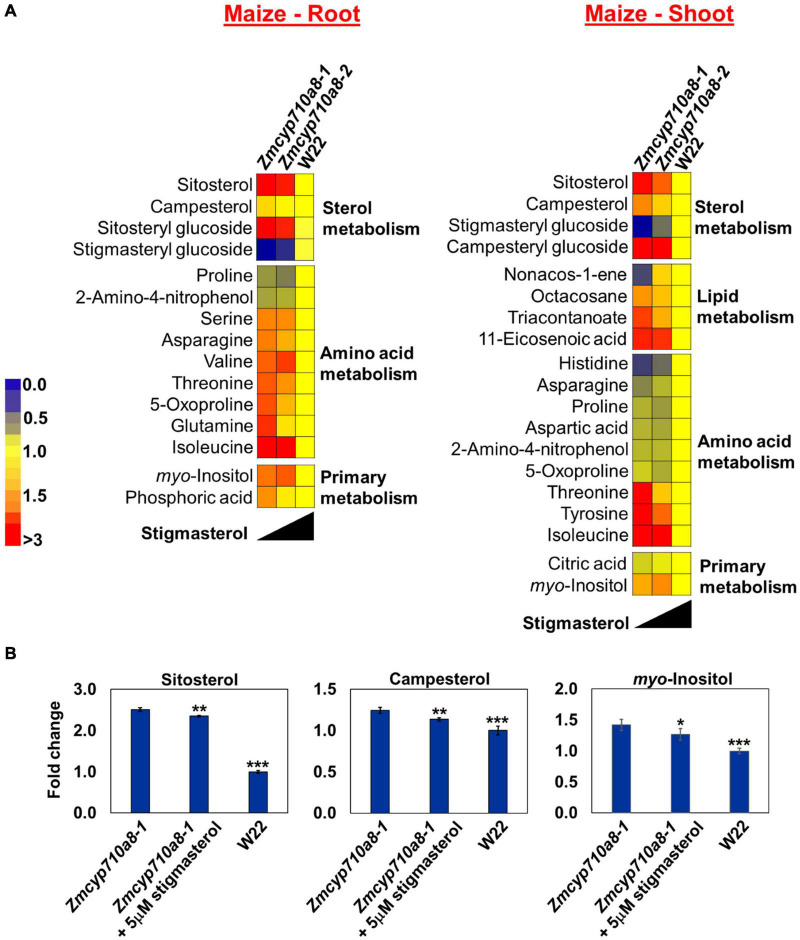
Stigmasterol modification impacts metabolite profile in root and shoot tissues of *Zmcyp710a8* mutants. **(A)** Non-targeted metabolite profile from roots and shoots of wild type and the Z*mcyp710a8* mutants were analyzed as described in section “Materials and Methods.” Relative metabolite levels in the two maize mutant alleles compared to their wild type are shown as fold changes in roots and shoots. The colors indicate the degree of fold change relative to wild type as shown in the color scale. **(B)** Fold changes of selected metabolites in the root tissue of *Zmcyp710a8-1* mutant allele with exogenous stigmasterol feeding. Asterisks indicate statistical significance by Students *t*-test compared to *Zmcyp710a8-1*. *P < 0.05; **P < 0.01; ***P < 0.001.

To identify cellular metabolites influenced by stigmasterol, we used non-targeted GC/MS metabolite analysis of roots and shoots of maize *Zmcyp710a8* and wild type. Molecular features with significant changes among maize genotypes were identified based on the filtering criteria as described in section “Materials and Methods.” A total of 29 molecular features (13 known and 16 unknown) were identified in roots and 31 features (17 known and 14 unknown) in shoots (Figure 7 and Supplementary Data 2). The concentration of molecular features correlated with stigmasterol content among genotypes in maize and the features belonged to similar classes including sterol, amino acid, primary metabolism, and unknown features in roots and shoots (Figure 7 and Supplementary Figure 7).

Metabolites such as glutamine and an unknown feature with retention index (RI) 1796.8 in *Zmcyp710a8-1* roots were 1.6- and 1.9-fold higher compared to W22, respectively (Figure 7A and Supplementary Figure 7). Threonine and tyrosine in shoots were 1.6- and 1.7-fold higher in *Zmcyp710a8-1* compared to W22 (Figure 7A), respectively. Among genotypes, changes in metabolites correlated with stigmasterol content (Figure 7A). For instance, threonine and *myo*-inositol showed a negative correlation with stigmasterol content in roots and shoots. Other metabolites that negatively correlated with stigmasterol were asparagine, 5-oxoproline and glutamine in roots, and tyrosine and triacontanoate in shoots (Figure 7A). Furthermore, unknown features with RI 1286.4 in roots and shoots; 1475.7, 1796.8, 1178.2, and 1600.6 in roots, and 2097.6 and 3074.5 in shoots were among the metabolites inversely correlated with stigmasterol (Supplementary Figure 7). Similarly, sitosteryl and campesteryl glucosides in roots and shoots, respectively, correlated negatively with stigmasterol (Figure 7A). Compounds that correlated positively with stigmasterol were stigmasteryl glucoside in roots and shoots and histidine, asparagine and non-acos-1-ene in shoots (Figure 7A). Unknown features with RIs of 1969.8 in roots and shoots; 2062.7, 1621.8, and 1720.0 in roots; 1545.3 and 1483.0 in shoots were also directly correlated with stigmasterol (Supplementary Figure 7).

Since changes in metabolites among the genotypes correlated with stigmasterol content, we reasoned that exogenous stigmasterol may reverse the metabolite levels in *Zmcyp710a8-1* mutant to wild type levels. Treatment of the *Zmcyp710a8-1* mutant with exogenous stigmasterol shifted the levels of sitosterol, campesterol, and *myo*-inositol toward W22 levels (Figure 7B), suggesting a complementation effect.

### Stigmasterol Impacts Global Cellular Metabolites in *Arabidopsis*

**FIGURE 8:**
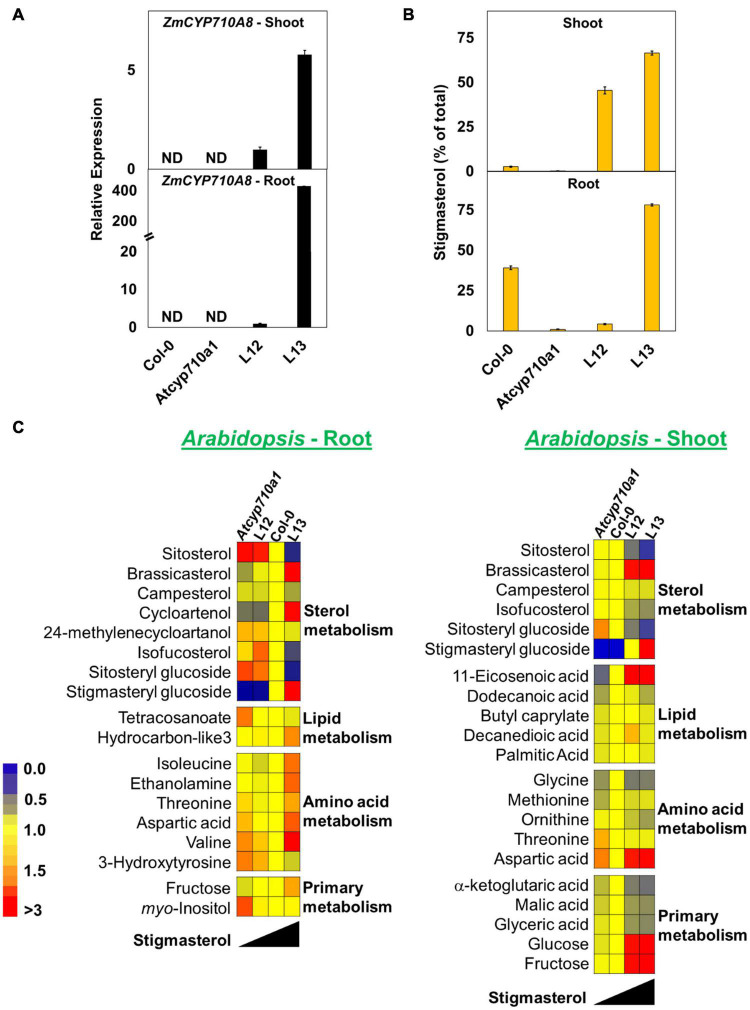
Stigmasterol modification impacts metabolite profile in root and shoot tissues of *Arabidopsis Atcyp710a1* expressing *ZmCYP710A8.* **(A)** Relative quantification of *ZmCYP710A8* mRNA in two independent *Arabidopsis* lines (L12 and L13) expressing the *35S:ZmCYP710A8* construct (Figure 2B) in *Atcyp710a1* background. Root and shoot tissue samples were separately pooled from >100 seedlings (3-week-old) grown in magenta boxes as described in section “Materials and Methods.” Data are means ± SD of three replicates. *AtEF1α-A* was used as a reference gene for normalization to be consistent with other occurrences. ND, not detected. **(B)** The composition (% of total sterols) of stigmasterol in root and shoot of Col-0, *Atcyp710a1* mutant and lines L12 and L13 (as in panel **A**) are shown. Tissue samples were pooled from >100 seedlings (3-week-old) grown in magenta boxes as described in section “Materials and Methods.” Roots and shoots were harvested separately from one box for a biological replicate. Data are means ± SD from three biological replicates. **(C)** Non-targeted metabolite profile from roots and shoots of Col-0, *Atcyp710a1* mutant and lines L12 and L13 were analyzed as described in section “Materials and Methods.” Relative metabolite levels compared to wild type are shown as fold changes in roots and shoots. The colors indicate the degree of fold change relative to wild type as shown in the color scale.

To test whether stigmasterol impacts cellular metabolism in a dicot species, we extended the study to *Arabidopsis*. The *Atcyp710a1* mutant (Wang et al., 2012) produces trace amounts of stigmasterol in roots (44.74 μg g^–1^ DW) and shoots (12.29 μg g^–1^ DW) providing a good system to test the impact of restoring or increasing stigmasterol production on cellular metabolism (Table 2). The full-length *ZmCYP710A8* ORF with the 35S viral promoter (Figure 2A) was transformed into *Atcyp710a1* allowing us to obtain several lines (data not shown) expressing *ZmCYP710A8*. Two lines (L12 and L13) with single insertion of the transgene in T4 generation (Supplementary Data 1) were selected for further analysis. There was a direct correlation between stigmasterol content and *ZmCYP710A8* mRNA in each of the transgenic lines (Figures 8A,B). In line L13, *ZmCYP710A8* mRNA and stigmasterol were high in roots (3417.28 μg g^–1^ DW) and shoots (2766.02 μg g^–1^ DW), while their sitosterol levels were reduced consistent with the presence of *At*CYP710A1 in Col-0 and *Zm*CYP710A8 in L13. In contrast, *ZmCYP710A8* mRNA and stigmasterol concentration of line L12 in roots (182.76 μg g^–1^ DW) and shoots (1824.12 μg g^–1^ DW) was reciprocal to that of the wild type (Figures 8A,B and Table 2). Line L12 had a higher sitosterol content in roots as in *Atcyp710a1* consistent with low *ZmCYP710A8* mRNA in L12 and reduced *At*CYP710A1 activity in *Atcyp710a1*. In roots and shoots, sitosterol and campesterol followed an inverse trend with stigmasterol while brassicasterol positively correlated with stigmasterol (Table 2). The campesterol content in shoots was also similar between *Atcyp710a1* and Col-0 but lower in L12 and L13. Brassicasterol levels were comparable among *Atcyp710a1*, Col-0 and L12 (69.44–95.96 μg g^–1^ DW) but were considerably higher in L13 (136.07 μg g^–1^ DW) (Table 2).

**TABLE 2:** Free sterols content (μg g^–1^ DW) in *Arabidopsis* seedlings.

Tissue	Free sterols	*Atcyp710a1*	L12	WT (Col-0)	L13
**Root**	Campesterol	502.81 ± 55.67	498.35 ± 44.94	592.99 ± 62.90	383.37 ± 40.46
	Brassicasterol	44.13 ± 11.25	67.33 ± 19.70	72.28 ± 8.62	230.06 ± 28.19
	Sitosterol	3612.07 ± 123.24	3493.94 ± 97.72	2165.25 ± 55.60	339.73 ± 18.37
	Stigmasterol*	44.74 ± 5.61	182.76 ± 11.78	1826.08 ± 129.59	3417.28 ± 128.40
	Total	4203.76 ± 191.29	4242.38 ± 136.35	4656.6 ± 201.53	4370.44 ± 165.5

		***Atcyp710a1***	**WT (Col-0)**	**L12**	**L13**

**Shoot**	Campesterol	585.30 ± 19.09	603.50 ± 5.78	529.78 ± 41.48	518.37 ± 12.78
	Brassicasterol	69.44 ± 17.08	72.76 ± 5.90	95.96 ± 15.47	136.07 ± 21.93
	Sitosterol	3301.29 ± 107.65	3371.78 ± 172.62	1553.63 ± 79.86	736.19 ± 58.47
	Stigmasterol*	12.29 ± 5.96	109.73 ± 13.44	1824.12 ± 156.02	2766.02 ± 171.34
	Total	3968.31 ± 146.3	4157.78 ± 154.21	4003.49 ± 232.39	4156.65 ± 217.06

^*^ Samples are organized based on stigmasterol concentration.

We also used non-targeted GC/MS analysis to study cellular metabolites in the *Atcyp710a1* mutant, Col-0, L12 and L13. Molecular features with significant changes among *Arabidopsis* genotypes were identified like in maize as described in section “Materials and Methods.” A correlation between *Arabidopsis* genotypes and the concentration of molecular features was observed. Metabolite concentrations among the *Arabidopsis* genotypes correlated with their stigmasterol content like maize (Figure 8C). A total of 39 molecular features (15 known and 24 unknown molecular features) were identified in roots and 28 features (17 known and 11 unknown molecular features) in shoots (Figure 8C and Supplementary Data 3). Sitosteryl glucoside levels in roots and shoots were negatively correlated with stigmasterol, whereas stigmasteryl glucoside was positively correlated with stigmasterol (Figure 8C). Methionine was 0.7-fold and RI 2288.9 was 2.4-fold in shoots of *Atcyp710a1* as in Col-0 (Figure 8C), and *myo*-inositol and threonine were affected in both *Arabidopsis* and maize shoots. It is noteworthy that, *Zmcyp710a8-1* and *Zmcyp710a8-2* have increased levels of several amino acids in roots and shoots. Conversely, the *Atcyp710a1* mutant produces comparable levels of several amino acids in roots and shoots, but there is an upward shift in amino acid levels when stigmasterol is increased as in L13 (Figure 8C).

Metabolites such as tetracosanoate and molecular feature RI 2996.5 were 1.4- and 1.6-fold higher, respectively, in *Atcyp710a1* roots compared to Col-0 and L13 (Figure 8C and Supplementary Figure 8). Several other metabolites were also affected in *Arabidopsis* correlating with stigmasterol, fructose and aspartic acid contents having negative correlations with stigmasterol in roots and shoots (Figure 8C). Other compounds that correlated negatively with stigmasterol in roots were 24-methylenecycloartanol, tetracosanoate, and several unknown features with retention indices (RI) 1743.4, 1853.8, 2430.4, 2686.9, 2815.5, 2996.5, 3313.5, 3436.2, 3574.1, and 3616.1 (Figure 8C and Supplementary Figure 8). Metabolites correlated positively with stigmasterol content in roots were cycloartenol, hydrocarbon-like3 (an unknown molecular feature with spectral similarity to known normal-chain hydrocarbons), isoleucine and 3-hydroxytyrosine, and unknown features with RIs 1496.8, 1578.5, and 2382.9 (Figure 8C and Supplementary Figure 8).

In shoots, isofucosterol, ornithine, α-ketoglutaric acid, malic acid, glyceric acid and a few unknown features with RIs 1965.6, 2288.9, and 2430.4 were inversely correlated with stigmasterol (Figure 8C and Supplementary Figure 8). On the other hand, there was a direct correlation between stigmasterol and several metabolites such as 11-eicosenoic acid, methionine, glucose, and unknown features with RI 1525.6, and 1945.7 in shoots (Figure 8C and Supplementary Figure 8).

## Discussion

Stigmasterol content fluctuates during development of plants such as maize (Kemp et al., 1967; Figure 3), sorghum (Heupel et al., 1986), pea (*Pisum sativum*, Schrick et al., 2011), and *N. benthamiana* (Suza and Chappell, 2016), and in response to environmental cues (Aboobucker and Suza, 2019). Further, stigmasterol content correlates with *LeCYP710A11* mRNA during tomato ripening (Whitaker and Gapper, 2008), suggesting that genes encoding sterol C-22 desaturases are responsive to developmental and environmental cues. Indeed, *ZmCYP710A8* expression is impacted by phytohormones (Figure 4), while treatment of tomato with abscisic acid (ABA) affected the expression of *LeCYP710A11* (García, 2019). In addition, *Meloidogyne incognita* infection suppressed *LeCYP710A11* (Cabianca et al., 2021), and *Pseudomonas syringae* attack increased the expression of *AtCYP710A* in *Arabidopsis* (Griebel and Zeier, 2010; Wang et al., 2012).

Multiple copies of sterol C-22 desaturase genes are found in *Arabidopsis* (Morikawa et al., 2006; Arnqvist et al., 2008), rice (Figure 1B), moss (Morikawa et al., 2009), and poplar (Arnqvist et al., 2008). In contrast, maize encodes a single sterol C-22 desaturase (Figures 1, 2, 3, 5). The strong correlation between *ZmCYP710A8* mRNA and stigmasterol suggests that control of stigmasterol production in maize occurs mainly at the transcriptional level.

We report here the identification of a maize stigmasterol mutant (Figure 5). To our knowledge, this is the first report of a maize mutant completely lacking the ability to produce an end product sterol. Yet the lack of stigmasterol does not result in any observable growth or developmental defects and mutant plants grow normally and produce seeds (Supplementary Figure 4). Like *Zmcyp710a8*, the tomato *Lecyp710a11* (García, 2019) and *Arabidopsis Atcyp710a1* (Morikawa et al., 2006; Senthil-Kumar et al., 2013; Figure 7B) stigmasterol mutants grow well and set seed in controlled environments. This suggests that stigmasterol plays a non-essential and potentially “vestigial” function in both monocot and dicot species. Perhaps, sitosterol compensates the bulk function of stigmasterol in the sterol C-22 desaturase mutants because sitosterol and campesterol are also involved in regulating membrane fluidity (Hartmann, 1998) and order (Grosjean et al., 2015).

Mass spectrometry imaging revealed that sitosterol is localized to the meristematic and elongation zone, suggesting elevated activity of ZmCYP710A8 might be responsible for the depletion of sitosterol in the differentiation zone (Figures 3B, 5F). Interestingly, the localization of sitosterol to the root tip correlates with localization of key sterol biosynthesis enzymes such as SMT1 and SQS (Devarenne et al., 2002; Willemsen et al., 2003), supporting the idea that metabolon formation occurs in sterol biosynthesis. This would also imply the localization at the same sites of the succeeding enzymes (Figure 1) all the way down to sterol-24-reductase to produce sitosterol. It is also noteworthy that stigmasterol concentration is reduced in the *smt1*^*orc*^ mutant, which displays mislocalization of PIN proteins involved in auxin transport (Betts and Moore, 2003; Willemsen et al., 2003). Therefore, the *Zmcyp710a8* mutant might also be a useful tool to study whether stigmasterol has a role in PIN protein distribution.

Because of lack or reduced activity of sterol C-22 desaturase activity, sitosterol content is elevated in *Zmcyp710a8* and *Atcyp710a1* mutants, yet the pool of ethyl sterols remained unaltered (Figure 3 and Table 2). The cross-species observations suggest that plants have a mechanism to maintain the ratio of ethyl and methyl sterols. Since stigmasterol modulates *ZmSMT2* mRNA (Figure 6), it might play a role in the maintenance of ethyl versus methyl sterols *via* SMT2. Indeed, the ratio of ethyl to methyl sterols is altered in *AtSMT2* and the *smt2/cvp1* mutant (Schaller et al., 1998; Schaeffer et al., 2001; Carland et al., 2002) but tobacco plants overexpressing *SMT2* cDNA produced more sitosterol without change in their stigmasterol content (Schaller et al., 1998).

Changes in steryl glucosides in both *Arabidopsis* and maize mutants were proportional to their free sterol precursor (Supplementary Figure 5). Yet, despite the abundance of sitosterol in maize and *Arabidopsis* stigmasterol mutants, their sitosteryl glucosides did not increase beyond the sitosterol content suggesting tight regulation of sterol glucosides. How steryl glycoside content is regulated to correlate with free sterols in maize stigmasterol mutants may provide insights to help understand sterol homeostasis in plants.

In contrast to animal systems, where mechanisms controlling cholesterol homeostasis are well known, the regulation of plant sterols remains a subject of interest. Supplying exogenous stigmasterol to *Zmcyp710a8* mutant allowed the interrogation of its impact on the expression of some of the key sterol biosynthesis genes. The positive effect of stigmasterol on *ZmHMGR* mRNA (Figure 6) contrasts with findings from mammalian systems where cholesterol has a negative effect on *HMGR* at the mRNA and enzyme levels (Goldstein and Brown, 1990). Nonetheless, feedback and feedforward transcriptional regulation of the sterol pathway was reported after the *NbCAS* gene was silenced in *N. benthamiana* (Atsumi et al., 2018). Perhaps, this was a response for silencing both cholesterol and phytosterol pathways because earlier findings suggested a feedback response to exogenous cholesterol in tobacco (Bhatt and Bhatt, 1984). Although we did not measure the enzymatic activities for HMGR and SMT2, the data (Figures 3, 6) suggest positive feedback by stigmasterol through a yet to be discovered (stigma)sterol sensing system (Aboobucker and Suza, 2019).

Several metabolites were altered in *Arabidopsis* and maize, and they correlated with stigmasterol content (Figures 7, 8). To our knowledge, this is the first study of the impact of stigmasterol on cellular metabolites. Although stigmasterol modification impacts similar classes of metabolites in both *Arabidopsis* and maize, there are differences in amino acid profiles (Figures 7, 8). The accumulation of several amino acids in the *Zmcyp710a8* lines (Figure 7) might present an opportunity for genetic improvement of the nutritional quality of maize. Studies are underway to measure amino acids in *Zmcyp710a8* seed.

As previously mentioned, amino acid and primary metabolism are among the metabolite classes impacted (Figures 7, 8), which might be expected because of their intricate connection. The products of glycolysis and citric acid cycle serve as precursors for amino acid biosynthesis and, in the case of energy deficiency, some amino acids including isoleucine are catabolized for energy production (Pratelli and Pilot, 2014; Yang et al., 2020). In addition to energy production, amino acid metabolism has other functions including growth and development, stress response, plant defense, nitrogen, and secondary metabolism (Pratelli and Pilot, 2014; Yang et al., 2020). Interestingly, exogenous sitosterol treatment in white clover modified amino acid content and water stress tolerance (Li et al., 2019), which might imply a potential conversion of stitosterol to stigmasterol to impart the observed changes.

Since isoleucine plays a role in abiotic stress response (Joshi et al., 2010), its elevation in the *Zmcyp710a8* background may be a form of stress compensation (Pratelli and Pilot, 2014). In addition, isoleucine is also elevated in the roots of L13 plants that overproduce stigmasterol. The data support earlier findings implicating stigmasterol in plant response to stress (Wang et al., 2012; Senthil-Kumar et al., 2013; Li et al., 2019). Moreover, *myo*-inositol, which is involved in the biosynthesis of ascorbate and abiotic stress response (Loewus and Murthy, 2000; Lorence et al., 2004) is also elevated in *Zmcyp710a8*, offering additional support of potential stress compensation. Therefore, the lack of observable phenotypes in *Zmcyp710a8* might be because stigmasterol is important only under certain situations to modulate membrane properties and/or act as a free molecule to signal specific response (Valitova et al., 2016; Aboobucker and Suza, 2019). Examples of the situations include pathogen resistance (Wang et al., 2012) and temperature stress tolerance (Senthil-Kumar et al., 2013). Plans are underway to perform detailed phenotypic characterization of the *Zmcyp710a8* mutant under stress conditions to help reveal characteristics that were not apparent in the present study.

In summary, the *Zmcyp710a8* mutant has enabled generation of data suggesting a role for stigmasterol in modulating cellular metabolism. Even though the metabolic function of stigmasterol might not be essential for growth and development under normal conditions, the *Zmcyp710a8* mutant is of great utility to interrogate many other biological and agronomic questions, such as gene expression studies, spatio-temporal localization of sterols and membrane proteins, cellular metabolism, pathway regulation, physiological studies, and crop improvement.

## Data Availability Statement

The datasets presented in this study can be found in online repositories. The names of the repository/repositories and accession number(s) can be found in the article/Supplementary Material.

## Author Contributions

WS conceived the research. WS and SA designed the research. SA, WS, and LS carried out the experiments and performed data analyses. All authors wrote the manuscript.

## Conflict of Interest

The authors declare that the research was conducted in the absence of any commercial or financial relationships that could be construed as a potential conflict of interest.

## Publisher’s Note

All claims expressed in this article are solely those of the authors and do not necessarily represent those of their affiliated organizations, or those of the publisher, the editors and the reviewers. Any product that may be evaluated in this article, or claim that may be made by its manufacturer, is not guaranteed or endorsed by the publisher.

## Footnotes

1
https://phytozome-next.jgi.doe.gov/

2
www.maizegdb.org
