# Supplementary material for: Maize Zmcyp710a8 Mutant as a Tool to Decipher the Function of Stigmasterol in Plant Metabolism
Source: Front Plant Sci. 2021 Nov 3;12:732216. doi: 10.3389/fpls.2021.732216 (PMC8597121; doi:10.3389/fpls.2021.732216)
Supplement: Supplementary file 4 [file Data_Sheet_1.docx]

Supplementary Material

**1. Supplementary Figures**

**
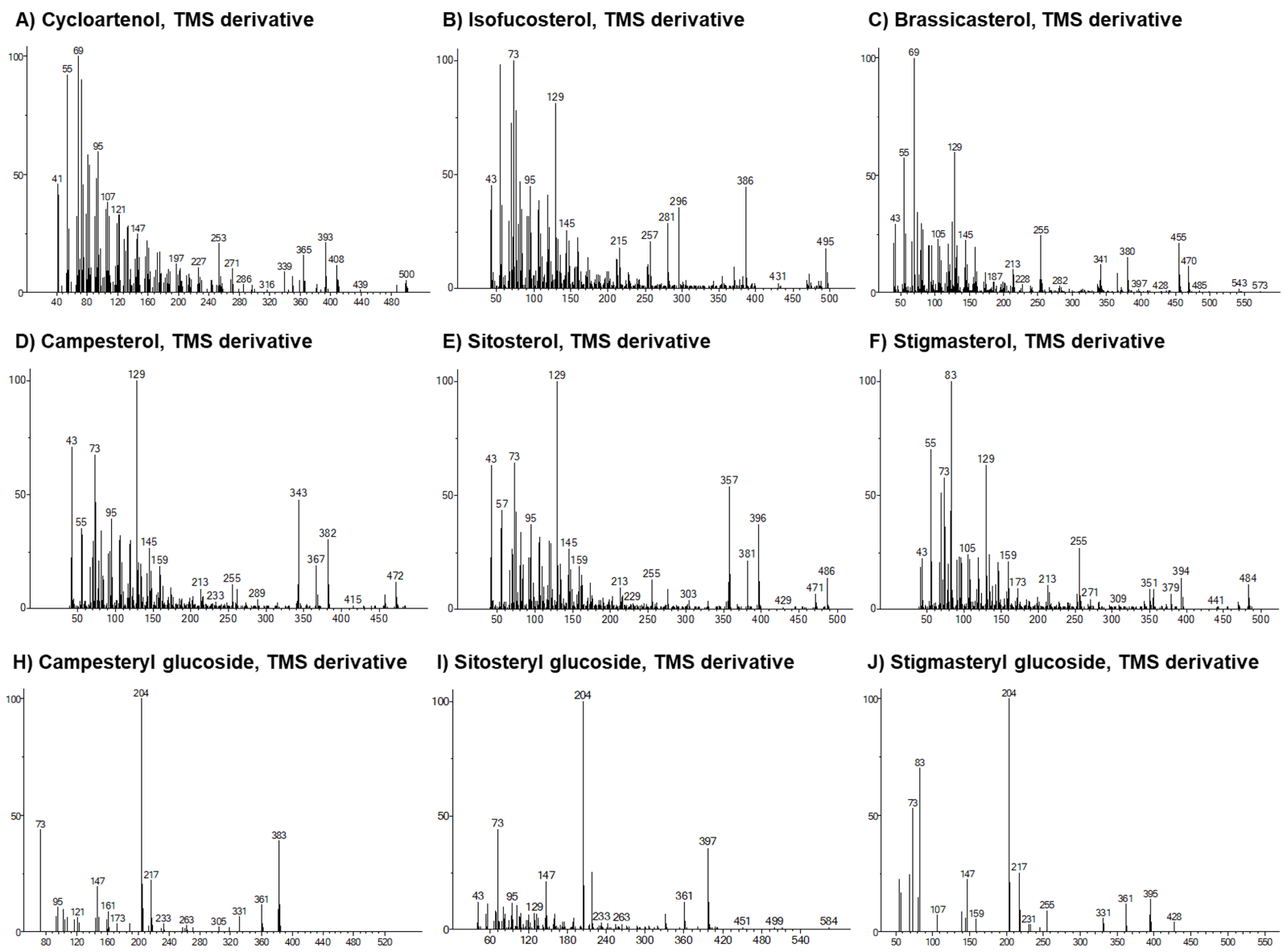
**

**Supplementary Figure 1.** MS spectra of sterols and steryl glucosides

MS spectra of sterol intermediates are in A) Cycloartenol, B Isofucosterol. Major sterols are in C) Brassicasterol, D) Campesterol, E) Sitosterol, F) Stigmasterol. Steryl glucosides as compared with (Phillips et al., 2005) are in H) Campesteryl glucoside, I) Sitosteryl gucoside and J) Stigmasteryl gucoside.


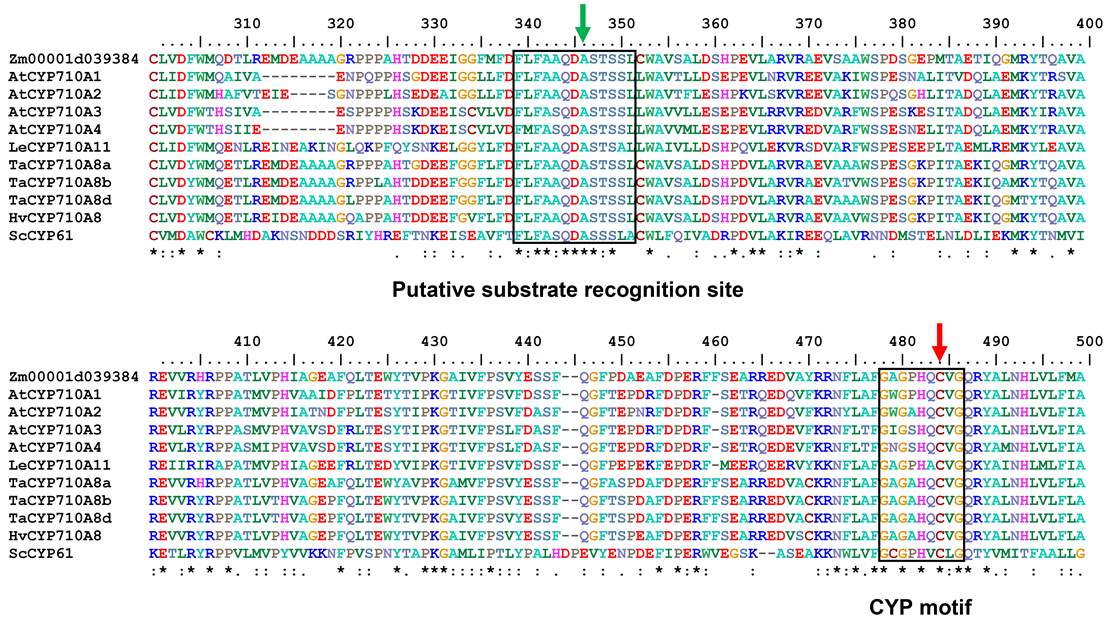


**Supplementary Figure 2.** Multiple sequence alignment of previously characterized CYP710A protein sequences and putative ZmCYP710A8 sequence (Zm00001d039384).

The sequence FLFA(A/S)QDAS(T/S)S, corresponding to a substrate recognition site (SRS4) of P450s (Gotoh, 1992), is boxed. The Ala-299 of AtCYP710A1, which is putatively involved in the desaturation reaction (Morikawa et al., 2006), is marked with a green arrow. The characteristic CYP motif is also boxed, and the heme ligand Cys residue is marked with a red arrow. The gene identifiers are as presented in (Aboobucker and Suza, 2019). Zm, *Zea mays*; Ta, *Triticum aestivum*; Hv, *Hordeum vulgare*; Le, *Lycopersicum esculentum*; At, *Arabidopsis thaliana*; Sc, *Saccharomyces cerevisiae*. *****, indicates fully conserved residue; **:**, indicates conservation between groups of strongly similar properties; and **.**, weakly similar properties.


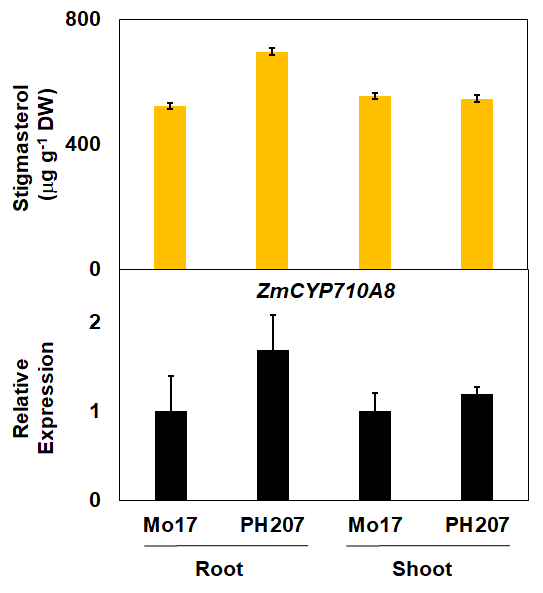


**Supplementary Figure 3.** Stigmasterol content (in μg g^-1^ DW) and quantification of *ZmCYP710A8* mRNA in different maize genotypes.

Seedlings were grown in cigar roll as previously described (Kumar et al., 2012) for 11 days without fungicide. Root and shoot tissues were pooled from 3 seedlings and used for sterol and mRNA quantification. *ZmACTIN* served as the reference gene for normalization. Data are means ± SD.

**Supplementary Figure 4.** Pictures of mature *Zmcyp710a8-1*, *Zmcyp710a8-2* and W22 plants grown side-by-side in Summer 2020 in Ag Engineering and Agronomy Research Farm, Boone, IA, USA, and the GPS coordinates are 42.02286, -93.77687.


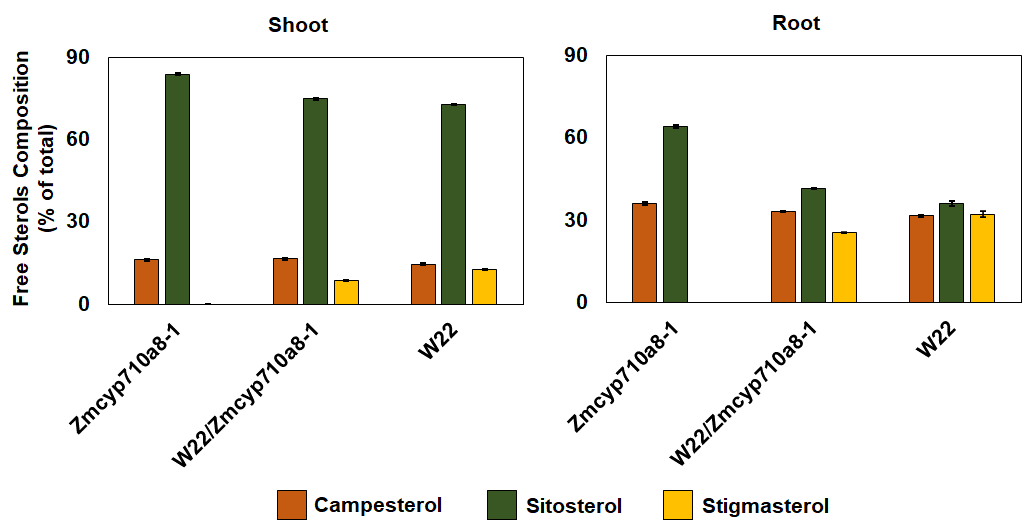


**Supplementary Figure 5.** The proportion (% of total sterols) of free major sterols in shoot and root of the null allele *Zmcyp710a8-1*, W22/*Zmcyp710a8-1* heterozygote and wild type (W22).

Tissue samples were pooled from 3 individual seedlings and used for sterol analysis. Data are means ± SD of three replicate measurements.


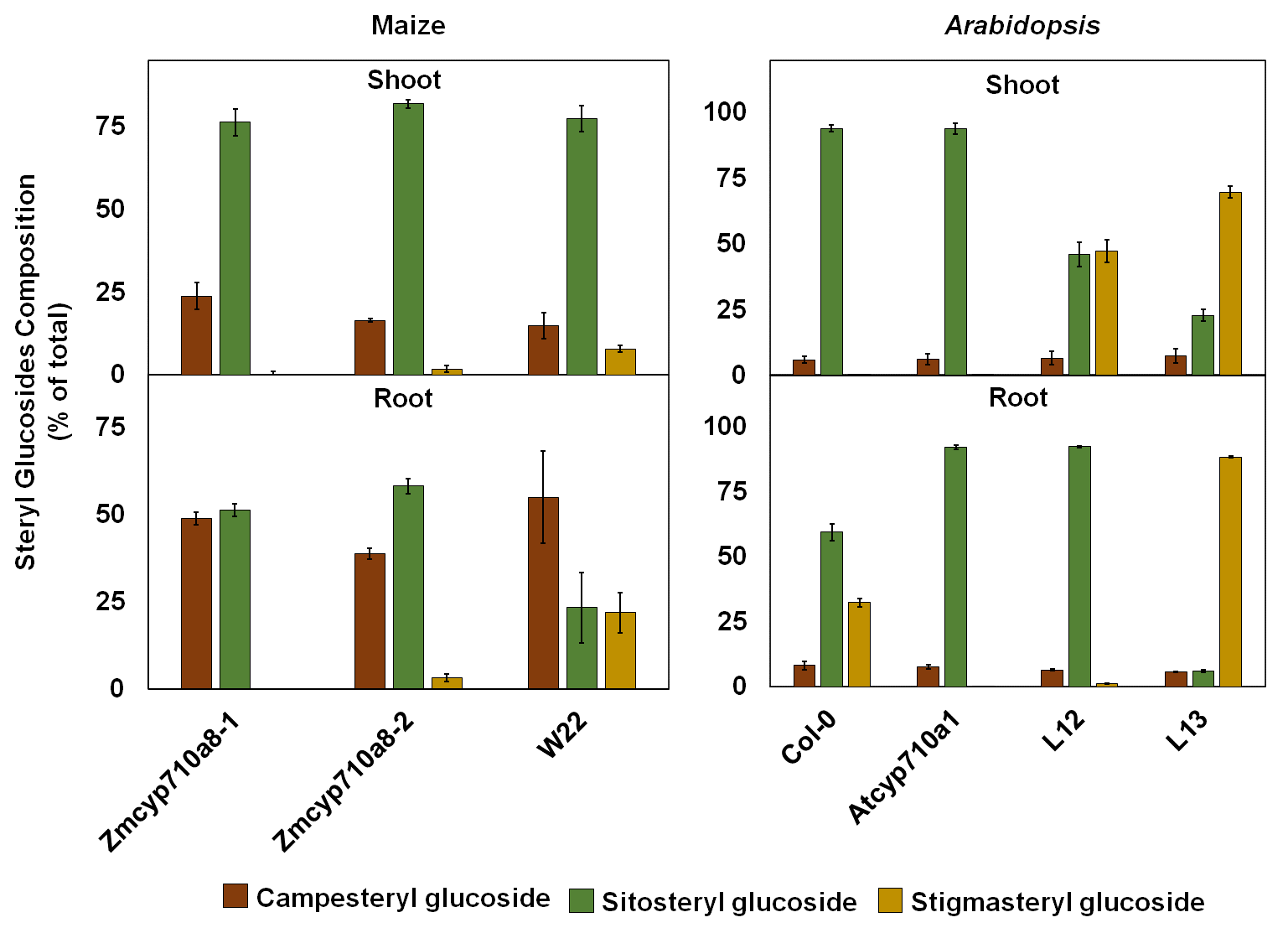


**Supplementary Figure 6.** Steryl glucosides composition (% of total steryl glucosides) in root and shoot of *Arabidopsis* and maize.

Tissue samples were pooled from 3 individual seedlings of maize *Zmcyp710a8-1*, *Zmcyp710a8-2* and W22 for maize. *Arabidopsis* tissue samples were pooled from >100 seedlings (3-week-old) grown in magenta boxes as described in “materials and methods” and used for sterol measurements. Data are means ±SD of three replicate measurements.


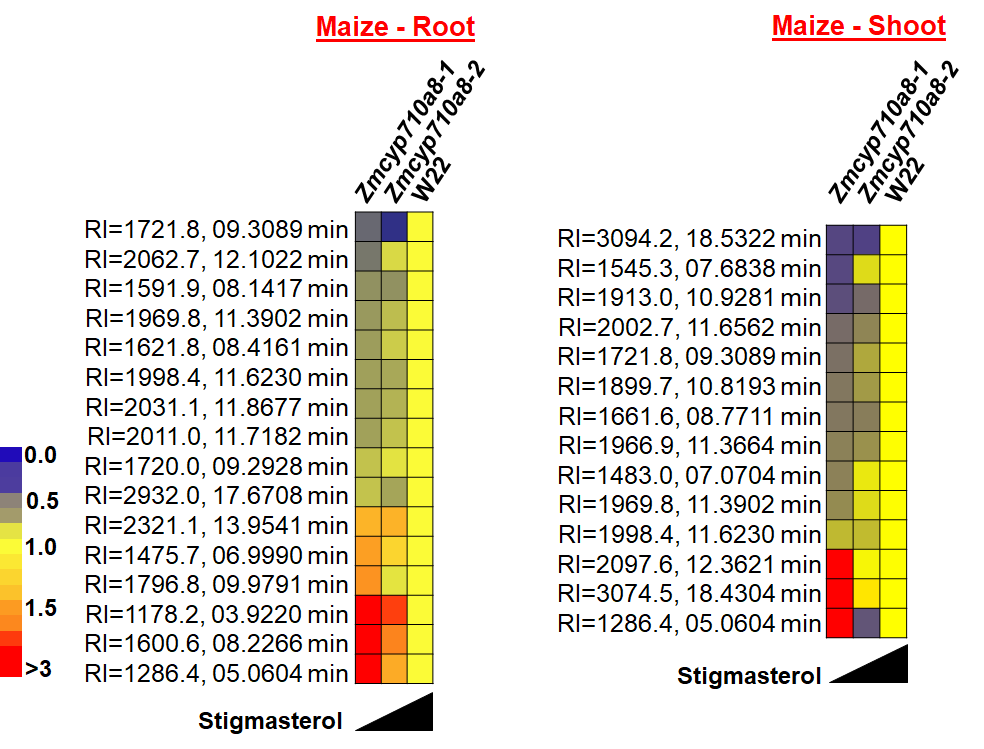


**Supplementary Figure 7.** Unknown metabolites impacted by stigmasterol modification in root and shoot tissues of *zmcyp710a8* mutants

Non-targeted metabolite profile from root and shoot of wild type and the *Zmcyp710a8* mutants were analyzed as described in “materials and methods”. Relative metabolite levels in the two maize mutant alleles compared to their wild type are shown as fold changes in Root and Shoot. The colors indicate the degree of fold change relative to wild type as shown in the color scale.


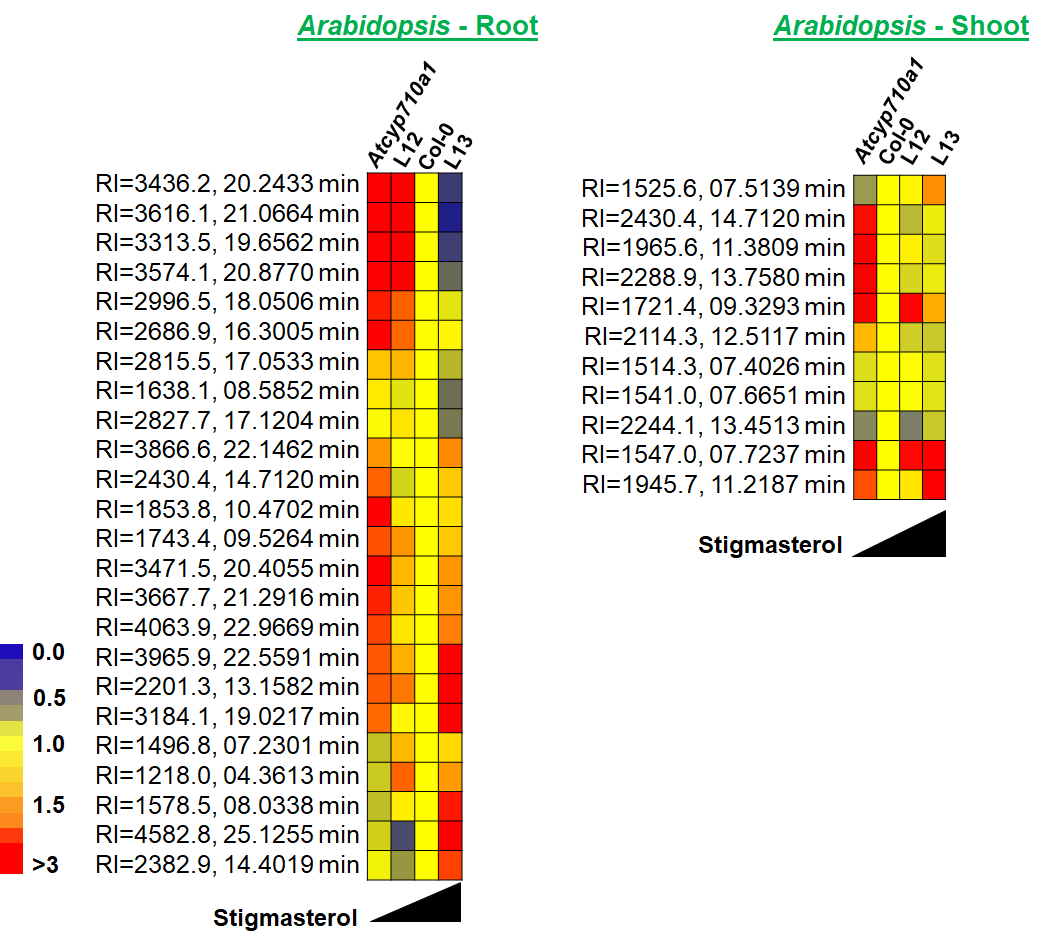


**Supplementary Figure 8.** Unknown metabolites impacted by stigmasterol modification in root and shoot tissues of *Arabidopsis*

Non-targeted metabolite profile from Root and Shoot of Col-0, *Atcyp710a1* mutant and lines L12 and L13 were analyzed as described in “materials and methods”. Relative metabolite levels compared to wild type are shown as fold changes in Root and Shoot. The colors indicate the degree of fold change relative to wild type as shown in the color scale.

**2. Supplementary Tables**

**Supplementary Table 1.** Primer sequences used in this study

| **Primer name** | **Primer sequence (5’ to 3’)** | **Purpose** |
| --- | --- | --- |
| ZmCYP710A8_F | GG***GGTACC***ATGGCGGCGCAGTCTCTGT | Cloning and transgene verification |
| ZmCYP710A8_R | CGC***GAGCTC***TCAGAACGACGGCAACTTGGC |  |
| Bar_F | CAGGAACCGCAGGAGTGGA | Transgene verification |
| Bar_R | CCAGAAACCCACGTCATGCC |  |
| 103F | GCGACATGAACCTGGAGACC | Genotyping transposon mutation in maize |
| 202R | GAGGAGGAGGCACGAGCAAG |  |
| TIR6^a^ | AGAGAAGCCAACGCCAWCGCCTCYATTTCGTC |  |
| qAtEF1a-A_F^b^ | TGAGCACGCTCTTCTTGCTTTCA | qRT-PCR |
| qAtEF1a-A_R^b^ | GGTGGTGGCATCCATCTTGTTACA |  |
| qZmACTIN_F^c^ | GGAGCTCGAGAATGCCAAGAGCAG |  |
| qZmACTIN_R^c^ | GACCTCAGGGCATCTGAACCTCTC |  |
| qZmCYP710A8_F | GCGACATGAACCTGGAGACC |  |
| qZmCYP710A8_R | CCGACGTTGAAGAGGTTGTAGTC |  |
| qZmHMGS_F | CCTCGCCATGGACATCTACT |  |
| qZmHMGS_R | CCAAGCCCAATGGTGTACTT |  |
| qZmHMGR_F | CTGGAGACTAGGCTGGGAGA |  |
| qZmHMGR_R | GATGGACGCGTAGTCGAAC |  |
| qZmSQS_F | CGACTGGCATTATTCGTGTG |  |
| qZmSQS_R | TCCAGGAAGGCAGTGGATAC |  |
| qZmSMT1_F | CTACCGTGTGCTGAAACCTG |  |
| qZmSMT1_F | CATCACCGAGCTCAATCTCA |  |
| qZmSMT2_F | TGGTGTACTGGTTCGTCTGG |  |
| qZmSMT2_R | ACTTGTCCTGCACCTTGTCC |  |
| qZmDWF1_F^d^ | GAGCAGTGGCTGATCGAGAAC |  |
| qZmDWF1_R^d^ | AACATGCGCCAGAAGTCCTT |  |

*KpnI* and *SacI* restriction sites in the forward and reverse primers, respectively, to facilitate cloning are in bold and italics. Previously reported primer sequences are: ^a^(Liu et al., 2016) ^b^(Aboobucker et al., 2017), ^c^(Louis et al., 2015), ^d^(Best et al., 2016)

**Supplementary Table 2.** Accession numbers of maize sterol biosynthesis genes described in this study and their *Arabidopsis* homologues

| **Gene** | **MaizeGDB Accession (B73 V4)** | ***Arabidopsis* homolog** |
| --- | --- | --- |
| *ZmHMGS* | *Zm00001d006166* |  |
|  | *Zm00001d048494* | *AtHMGS (At4g11820)* |
|  | *Zm00001d027383* |  |
| *ZmHMGR*^a^ | *Zm00001d030595 (ZmHMGR1)* | *AtHMGR1 (At1g76490)*  *AtHMGR2 (At2g17370)* |
|  | *Zm00001d006040 (ZmHMGR2)* |  |
|  | *Zm00001d050972 (ZmHMGR3)* |  |
|  | *Zm00001d051626 (ZmHMGR4)* |  |
|  | *Zm00001d052903 (ZmHMGR5)* |  |
|  | *Zm00001d017826 (ZmHMGR6)* |  |
|  | *Zm00001d020963 (ZmHMGR7)* |  |
| *ZmSQS* | *Zm00001d013048* | *AtSQS1 (At4g34640)* |
|  | *Zm00001d034516* | *AtSQS2 (At4g34650)* |
| *ZmSMT1* | *Zm00001d019139* |  |
|  | *Zm00001d013035* | *AtSMT1 (At5g13710)* |
|  | *Zm00001d013037* |  |
| *ZmSMT2* | *Zm00001d048356* | *AtSMT2-1 (At1g20330)* |
|  | *Zm00001d027548* | *AtSMT2-2 (At1g76090)* |
| *ZmDWF1*^b^ | *Zm00001d014887* | *AtDWF1 (At3g19820)* |
| *ZmCYP710A8* | *Zm00001d039384* | *AtCYP710A1 (At2g34500)*  *AtCYP710A2 (At2g34490)*  *AtCYP710A3 (At2g28850)*  *AtCYP710A4 (At2g28860)* |

Previously reported maize sterol biosynthetic genes are ^a^(Li et al., 2014), ^b^(Best et al., 2016)

**Supplementary Table 3.** Free sterol content (in μg g^-1^ DW) in shoot and root of various maize genotypes at V1 stage

|  |  | **B73** | **W22** | **PHB47** | **PHZ51** | **PHB47/PHZ51** |
| --- | --- | --- | --- | --- | --- | --- |
| **Shoot** | Sitosterol | 852.7 ± 25.5 | 967.6 ± 21.4 | 864.5 ± 26.6 | 846.4 ± 36.3 | 955.7 ± 69.9 |
|  | Stigmasterol | 332.1 ± 16.7 | 303.7 ± 14.8 | 231.9 ± 4.0 | 283.4 ± 10.0 | 288.6 ± 28.6 |
|  | Campesterol | 295.0 ± 16.6 | 274.7 ± 10.7 | 224.3 ± 3.5 | 264.2 ± 14.0 | 314.0 ± 23.6 |
|  | Total | 1479.8 ± 34.7 | 1546.0 ± 28.1 | 1320.6 ± 27.1 | 1394.1 ± 40.1 | 1558.3 ± 79.1 |
| **Root** | Sitosterol | 490.0 ± 25.0 | 659.4 ± 67.4 | 551.7 ± 7.1 | 442.9 ± 19.8 | 544.4 ± 21.3 |
|  | Stigmasterol | 1253.0 ± 44.2 | 989.5 ± 86.7 | 993.4 ± 16.1 | 1249.5 ± 52.5 | 1098.5 ± 26.1 |
|  | Campesterol | 542.4 ± 39.8 | 754.0 ± 74.0 | 529.3 ± 13.1 | 557.2 ± 48.8 | 591.1 ± 21.0 |
|  | Total | 2285.2 ± 64.5 | 2402.9 ± 132.4 | 2074.4 ± 21.9 | 2249.6 ± 74.3 | 2233.9 ± 39.7 |

Cholesterol was detected only in trace levels in all the samples. Tissue samples were pooled from 3 individual seedlings in one experiment and the data are means ± SD.

**Supplementary Table 4.** Fold changes of sterols in maize mutant alleles compared to wild type

| **Tissue** | **Sterols** | ***Zmcyp710a8-1*** | ***Zmcyp710a8-2*** |
| --- | --- | --- | --- |
| **Root** | Cycloartenol | 1.08 ± 0.34 ^ns^ | 0.75 ± 0.02 ^***^ |
|  | Isofucosterol | 0.89 ± 0.09 ^ns^ | 0.81 ± 0.11 ^*^ |
|  | Campesterol | 1.24 ± 0.07 ^*^ | 1.1 ± 0.03 ^*^ |
|  | Sitosterol | 2.51 ± 0.1 ^***^ | 2.34 ± 0.02 ^***^ |
|  | Stigmasterol | ND | 0.3 ± 0.01 ^***^ |
| **Shoot** | Cycloartenol | 1.02 ± 0.27 ^ns^ | ND |
|  | Isofucosterol | 1.04 ± 0.18 ^ns^ | 0.54 ± 0.02 ^***^ |
|  | Campesterol | 1.26 ± 0.04 ^***^ | 1.1 ± 0.02 ^***^ |
|  | Sitosterol | 1.53 ± 0.08 ^***^ | 1.34 ± 0.02 ^***^ |
|  | Stigmasterol | ND | 0.5 ± 0.01 ^***^ |

* P < 0.05, *** P < 0.001, ns – not significant by Student’s *t* test compared to W22. ND – Not detected. Tissue samples were pooled from 3 individual seedlings of *Zmcyp710a8-1*, *Zmcyp710a8-2* and W22 and used for sterol measurements. Data are means ± SD from three replicate measurements.

**3. References**

Aboobucker, S.I., and Suza, W.P. (2019). Why do plants convert sitosterol to stigmasterol? *Frontiers in Plant Science* 10**,** 354. doi: 10.3389/fpls.2019.00354.

Aboobucker, S.I., Suza, W.P., and Lorence, A. (2017). Characterization of two *Arabidopsis* L-gulono-1,4-lactone oxidases, AtGulLO3 and AtGulLO5, involved in ascorbate biosynthesis. *Reactive Oxygen Species* 4(12)**,** 389-417. doi: 10.20455/ros.2017.861.

Best, N.B., Hartwig, T., Budka, J., Fujioka, S., Johal, G., Schulz, B., et al. (2016). *nana plant2* encodes a maize ortholog of the *Arabidopsis* brassinosteroid biosynthesis gene *DWARF1*, identifying developmental interactions between brassinosteroids and gibberellins. *Plant Physiology* 171(4)**,** 2633-2647. doi: 10.1104/pp.16.00399.

Gotoh, O. (1992). Substrate recognition sites in cytochrome P450 family 2 (CYP2) proteins inferred from comparative analyses of amino acid and coding nucleotide sequences. *The Journal of Biological Chemistry* 267(1)**,** 83-90.

Kumar, B., Abdel-Ghani, A.H., Reyes-Matamoros, J., Hochholdinger, F., and Lübberstedt, T. (2012). Genotypic variation for root architecture traits in seedlings of maize (*Zea mays* L.) inbred lines. *Plant Breeding* 131(4)**,** 465-478. doi:  10.1111/j.1439-0523.2012.01980.x.

Li, W., Liu, W., Wei, H., He, Q., Chen, J., Zhang, B., et al. (2014). Species-specific expansion and molecular evolution of the 3-hydroxy-3-methylglutaryl coenzyme A reductase (HMGR) gene family in plants. *PLoS One* 9(4)**,** e94172. doi: 10.1371/journal.pone.0094172.

Liu, P., McCarty, D.R., and Koch, K.E. (2016). Transposon mutagenesis and analysis of mutants in UniformMu maize (*Zea mays*). *Current Protocols in Plant Biology* 1(3)**,** 451-465.

Louis, J., Basu, S., Varsani, S., Castano-Duque, L., Jiang, V., Williams, W.P., et al. (2015). Ethylene contributes to *maize insect resistance1*-mediated maize defense against the phloem sap-sucking corn leaf aphid. *Plant Physiology* 169(1)**,** 313-324. doi: 10.1104/pp.15.00958.

Morikawa, T., Mizutani, M., Aoki, N., Watanabe, B., Saga, H., Saito, S., et al. (2006). Cytochrome P450 CYP710A encodes the sterol C-22 desaturase in *Arabidopsis* and tomato. *The Plant Cell* 18(4)**,** 1008-1022. doi: 10.1105/tpc.105.037012.

Phillips, K.M., Ruggio, D.M., and Ashraf‐Khorassani, M. (2005). Analysis of steryl glucosides in foods and dietary supplements by solid-phase extraction and GC. *Journal of Food Lipids* 12(2)**,** 124-140. doi: 10.1111/j.1745-4522.2005.00011.x.
